# Genomic and transcriptomic analyses of *Heteropoda venatoria* reveal the expansion of P450 family for starvation resistance in spiders

**DOI:** 10.1093/gigascience/giaf019

**Published:** 2025-03-21

**Authors:** Guoqing Zhang, Yiru Wang, Hongcen Jiang, Yi Wang

**Affiliations:** Integrative Science Center of Germplasm Creation in Western China (CHONGQING) Science City, Biological Science Research Center, Southwest University, Chongqing 400715, China; Integrative Science Center of Germplasm Creation in Western China (CHONGQING) Science City, Biological Science Research Center, Southwest University, Chongqing 400715, China; Integrative Science Center of Germplasm Creation in Western China (CHONGQING) Science City, Biological Science Research Center, Southwest University, Chongqing 400715, China; Integrative Science Center of Germplasm Creation in Western China (CHONGQING) Science City, Biological Science Research Center, Southwest University, Chongqing 400715, China

**Keywords:** starvation resistance, *Heteropoda venatoria*, gene family expansion, cytochrome P450, transcriptomics

## Abstract

**Background:**

Research on the mechanism of starvation resistance can help reveal how animals adjust their physiology and behavior to adapt to the uncertainty of food resources. A low metabolic rate is a significant characteristic of spider physiological activity and can increase spider starvation resistance and adapt to complex ecological environments.

**Results:**

We sequenced the genome of *Heteropoda venatoria* and discovered significant expansions in gene families related to lipid metabolism, such as cytochrome P450 and steroid hormone biosynthesis genes, through comparative genomic analysis. We also systematically analyzed the gene expression characteristics of *H. venatoria* at different starvation resistance stages and reported that the fat body plays a crucial role during starvation in spiders. This study indicates that during the early stages of starvation, *H. venatoria* relies on glucose metabolism to meet its energy demands. In the middle stage, gene expression stabilizes, whereas in the late stage of starvation, pathways for fatty acid metabolism and protein degradation are significantly activated, and autophagy is increased, serving as a survival strategy under extreme starvation. Notably, analysis of expanded P450 gene families revealed that *H. venatoria* has many duplicated CYP3 clan genes that are highly expressed in the fat body, which may help maintain a low-energy metabolic state, allowing *H. venatoria* to endure longer periods of starvation. We also observed that the motifs of P450 families in *H. venatoria* are less conserved than those in insects are, which may be related to the greater polymorphism of spider genomes.

**Conclusions:**

This research not only provides important genetic and transcriptomic evidence for understanding the starvation mechanisms of spiders but also offers new insights into the adaptive evolution of arthropods.

## Introduction

Spiders, as widely distributed arthropods, possess remarkable survival abilities and occupy a unique ecological niche in nature [1]. They play dual roles in the food chain as both predators and prey, which underscores their critical importance in ecological systems [2]. For a long time, the ability of spiders to spin silk and inject venom has been the main feature of interest [3–8], particularly their unique ability to produce up to 7 distinct types of silk, an unmatched feat in nature [3, 9].

In addition to their predatory tactics involving silk spinning and venom injection, spiders have evolved robust starvation resistance to cope with unstable food supplies. Spiders typically adapt to a sedentary lifestyle, waiting for prey while remaining largely motionless, which highlights the significance of the resting metabolic rate throughout their life cycle [10]. Food supply constraints significantly shape the ecology and behavior of spiders, leading to relatively low metabolic rates [11]. The presence of tracheae plays a significant role in spiders, which have well-developed tracheal systems, as most spiders exhibit metabolic rates far below what would be expected on the basis of their body weight, especially those with 2 pairs of lungs [12]. Other factors, such as sex, life span, reproduction, developmental status, type of prey captured, and high anaerobic energy acquisition capabilities, also significantly influence resting and active metabolic rates. For example, spiders with a life span exceeding 1 year have lower metabolic rates than those with a 1-year life cycle [12, 13]. Energy homeostasis is achieved by balancing energy expenditure and energy intake. Cellular autophagy is a self-degradative process that is crucial for maintaining energy homeostasis during periods of starvation [14–16]. Research has shown that the evolution of social spiders is linked to nutritional metabolism and autophagy, which regulate metabolic processes and mitigate the threat of cannibalism to ensure an adequate energy supply [17].

Starvation resistance is an adaptive trait evolved by organisms to survive in environments with food scarcity. The starvation resistance of spiders allows them to extend their survival time under conditions of prey scarcity through various physiological mechanisms, such as reducing metabolic rates, decreasing activity levels, and utilizing stored energy [18, 19]. Additionally, the starvation resistance of spiders may be associated with specific behavioral adaptations, such as alterations in predation strategies, optimization of energy allocation, and improvement in the timing of reproductive investment [18]. The starvation resistance of spiders, as a core component of their survival strategy, not only directly impacts individual survival rates but also profoundly influences energy flow and material cycling within ecosystems [18]. As global climate change accelerates and habitat fragmentation intensifies, understanding how spiders adapt their physiological and behavioral strategies to cope with the unpredictability of food resources is crucial for predicting ecosystem responses and adaptability. Research on plant stress resistance is plentiful and has focused primarily on drought resistance, salt tolerance, chilling tolerance, and other biotic stresses [20–24]. In contrast, studies on animal stress resistance are relatively rare, and the starvation resistance of spiders is highly important in research on ecological adaptation. With the continuous advancement of biological research methods and the development of sequencing technologies [25, 26], we have the opportunity to investigate the mechanisms of spider starvation resistance from molecular, physiological, and behavioral ecological perspectives, as well as the ecological and evolutionary significance of this phenomenon.

*Heteropoda venatoria* (NCBI:txid152925) is a hunting spider characterized by well-developed limbs and extremely fast movement. *H. venatoria* does not spin webs; it is known for hunting live insects with exceptional agility and speed during the night. Additionally, the average life span of male *H. venatoria* is 465 days, whereas females live for approximately 580 days [27], both of which clearly surpass 1 year; these spiders can therefore be classified as spiders with a low metabolic rate [12]. Our observations revealed that *H. venatoria* has an extraordinary ability to endure starvation and is capable of surviving for more than 4 months without food as long as the humidity is maintained. However, there is very little research on the starvation endurance of spiders. To date, research on *H. venatoria* has focused mainly on its venom [28–31], and there is no high-quality genome sequence available for further exploration of the molecular mechanisms underlying its starvation resistance. A high-quality genome sequence is crucial for understanding how *H. venatoria* regulates its metabolism and survival under starvation conditions. With a genome, we can more effectively identify key genes and regulatory elements, thereby revealing genes and molecular pathways associated with starvation resistance; this helps us gain a deeper understanding of the underlying mechanisms involved. Therefore, this study aimed to investigate the expression of functional genes associated with starvation resistance in *H. venatoria* through genomic and transcriptomic data, explore its response strategies to environmental changes, and outline future research directions, with the goal of providing a scientific basis for the conservation of biodiversity and the maintenance of ecosystem functions.

## Results

### High-quality genome assembly and annotation of *H. venatoria*

The female *H. venatoria* spider has 22 chromosomes in its haploid set (2n = 44) [32]. To assess the complexity of the *H. venatoria* genome, we initially sequenced the female *H. venatoria* genome via Illumina technology and obtained approximately 184 Gb of raw data. *K*-mer analysis revealed that the genome size was 5.37 Gb, with a repeat proportion of 46.3% and a heterozygosity rate of 0.96% (Supplementary Fig. S1C).

**Figure 1:**
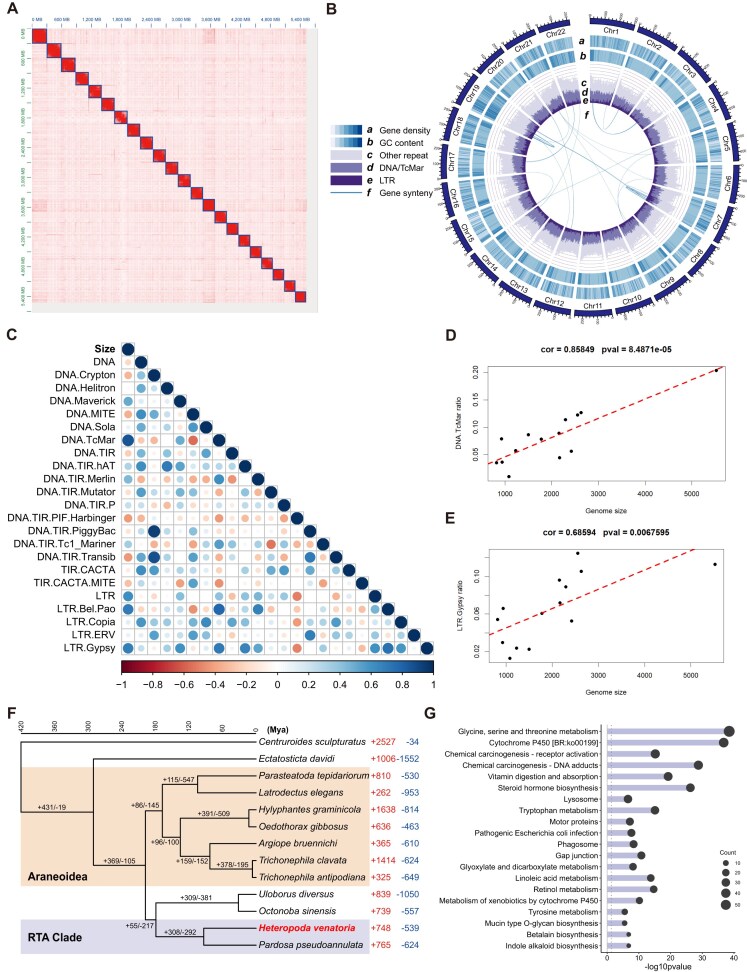
Chromosomal-scale genome assembly and genomic characteristics of *Heteropoda venatoria*. (A) Hi-C assembly map of *H. venatoria*. (B) Circular diagram depicting the genomic features of *H. venatoria*. (C) Correlations between genome size and the prevalence of different types of repeats. (D, E) Linear relationships of DNA. TcMar and LTR. Gypsy with genome size. (F) Phylogenetic tree of a scorpion and 12 spider species, along with the contraction and expansion of gene families. (G) KEGG functional enrichment of the expanded gene families in *H. venatoria*.

To achieve high-quality genome assembly for *H. venatoria*, we employed HiFi sequencing, which resulted in approximately 127 Gb of raw sequencing data. The initial assembly yielded a 5.95-Gb contig genome, with an N50 of 2.4 Mb. Using Hi-C for contig mounting, we ultimately obtained a genome consisting of 22 chromosomes, with a scaffold N50 of 253.94 Mb and a genome size of 5.52 Gb (Table 1), which was closely aligned with the 5.37 Gb obtained via a genome survey. The Hi-C map clearly demonstrated high continuity in the chromosome assembly (Fig. 1A). BUSCO analysis revealed an assembly completeness of 96.3%, with only 4.0% duplicated BUSCOs (Table 2), indicating that the high-quality genome assembly was suitable for subsequent analyses.

**Table 1:** Characteristics of the *Heteropoda venatoria* genome assembly

Estimated genome size (bp)	5,366,399,622
Heterozygosity (%)	0.96
Length of genome assembly (bp)	5,521,825,687
N50 of scaffolds (bp)	253,943,846
N50 of contigs (bp)	2,391,191
GC content (%)	35.07
Complete BUSCOs^a^(%)	96.3
Number of genes	31,547
Average gene length	45,334
Repeat content (%)	63.58

a BUSCO analysis was based on the metazoan lineage of protein-coding genes.

**Table 2:** BUSCO analysis of the *Heteropoda venatoria* genome assembly and annotation

	Genome assembly	Genome annotation
Complete BUSCOs^a^(%)	96.30%	94.30%
Complete and single-copy BUSCOs	92.30%	90.60%
Complete and duplicated BUSCOs	4.00%	3.70%
Fragmented BUSCOs	1.70%	1.50%
Missing BUSCOs	2.00%	4.20%

a BUSCO analysis was based on the metazoan lineage of protein-coding genes.

In terms of genome annotation, we identified 31,547 genes with an average length of 45 kb (Table 1), achieving a completeness rate of 94.3%. The functional annotations included Gene Ontology (GO) terms for 13,857 genes and KEGG pathways for 9,488 genes. With respect to repeat annotation, repeat sequences accounted for 63.58% of the genome (Table 1), which is significantly greater than the proportion of repeats estimated by *k*-mer analysis (Supplementary Fig. S1C). The impact of repeat sequences on genome size is significant [33–37]. Given the relatively high genome size and proportion of repeat sequences in *H. venatoria* compared with those in other spiders, we additionally collected genomic data from 12 other spider species and one scorpion to identify their repeat sequences. Analysis of repeat sequences across 14 spider genomes revealed a strong correlation between genome size and the presence of TcMar and LTR elements (Fig. 1C–E). Notably, these 2 types of repeat sequences also had the highest prevalence in *H. venatoria*. Interestingly, we found that in *H. venatoria* chromosomes, regions with a high proportion of repeats also presented an increase in GC content (Fig. 1B). We further quantified the GC content of the repeat region and the nonrepeat region and found that the GC content of the whole repeat region was significantly greater than that of the nonrepeat region (Supplementary Fig. S1D).

### Gene family expansion and contraction

We gathered genomic data and annotations for 1 scorpion and 11 chromosome-level spider genomes using the scorpion as an outgroup [38–49]. Using the maximum likelihood method, we constructed a phylogenetic tree encompassing these 12 arachnid species. Phylogenetic analysis revealed that *H. venatoria* is a member of the RTA clade, which is consistent with recent research findings [50]. Additionally, through the application of CAFE5 for the analysis of gene family expansion and contraction, we found that *H. venatoria* has expanded to a total of 748 gene families (Fig. 1F and Supplementary Table S4). To elucidate the functional implications of these expanded families, we performed functional enrichment analysis on the genes associated with those families that had undergone significant expansion. Our findings revealed that pathways related to lipid metabolism, including cytochrome P450 [BR:ko00199], steroid hormone biosynthesis, and linoleic acid metabolism, were significantly enriched in *H. venatoria* (Fig. 1G). We speculate that the formidable starvation tolerance of *H. venatoria* may be associated with the expansion of gene families related to lipid metabolism pathways within its genome.

### Transcriptome design for starvation resistance in *H. venatoria*

**Figure 2:**
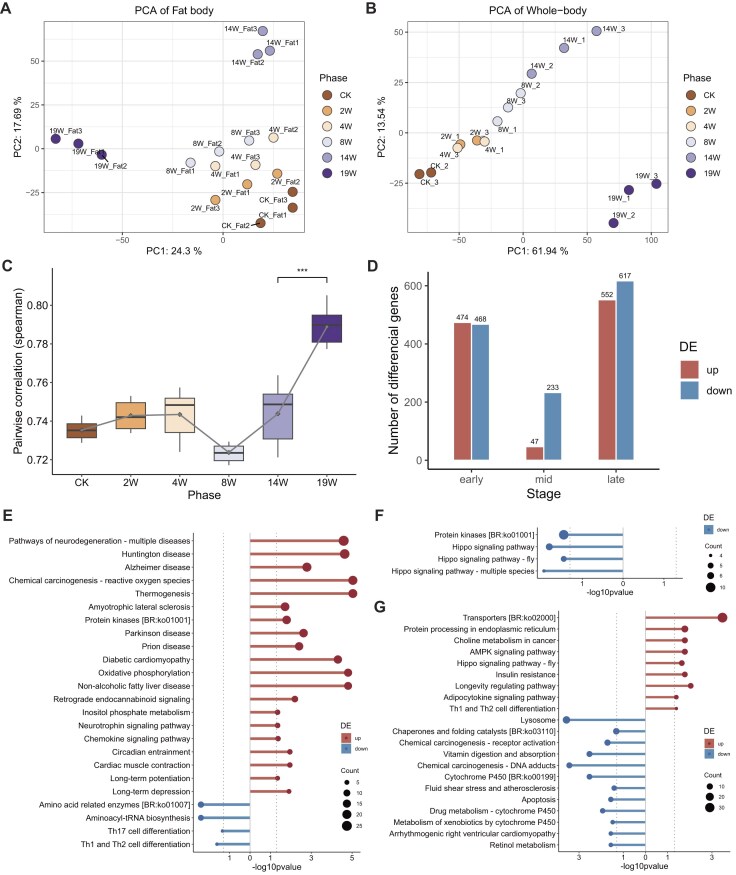
Transcriptomic analysis results of the fat body and whole-body responses to starvation resistance in *Heteropoda venatoria*. (A, B) PCA results of the starvation resistance-related transcriptome. (C) Correlation analysis of the transcriptomes of the fat body and whole body across various stages of starvation resistance. (D) Number of DEGs in the fat body transcriptome during the early, middle, and late stages of starvation resistance. (E, F, G) KEGG functional enrichment results for DEGs in the fat body transcriptome during the early, middle, and late stages of starvation resistance.

To further investigate the reasons behind the exceptional starvation resistance of *H. venatoria*, the *H. venatoria* samples subjected to starvation treatments were divided into 6 groups according to the duration of treatment. Principal component analysis (PCA) revealed that the expression profiles of the fat body transcriptome were more closely correlated with the duration of starvation in *H. venatoria* (Fig. 2A), whereas the whole-body transcriptome showed some overlap between different treatments (Supplementary Fig. S2A), likely due to the inclusion of numerous tissues, which resulted in tissue-specific variations overshadowing treatment effects. After abnormal samples were removed from the whole-body transcriptome, the results revealed that, except the 14- and 19-week samples, which presented obvious differences from the other samples, the remaining samples presented relatively minor expression variations (Fig. 2B).

According to the PCA of fat body expression, the samples were clustered into 4 distinct groups (Fig. 2A). Notably, the 4- and 8-week samples clustered closely, with the CK and 2-week samples being relatively proximal, whereas the 14- and 19-week samples were markedly divergent. We hypothesize that 14 and 19 weeks represent the later stages of starvation, when *H. venatoria*’s greatest gene activity is heightened, leading to greater transcriptomic differences in these samples. For subsequent analysis, we divided the starvation process into 3 phases: early starvation (CK and 2 weeks), middle starvation (4 and 8 weeks) and late starvation (14 and 19 weeks). Both fat body and whole-body expression analyses revealed that the 19-week samples were obviously different from the other samples (Fig. 2A, B). Therefore, in addition to the 3 main phases, the 19-week samples were analyzed separately.

### Differential transcriptomic analysis during the early, middle, and late stages of starvation in *H. venatoria*

In our study of the fat body transcriptome of *H. venatoria* during 3 distinct stages of starvation (early, middle, and late), we observed the following expression patterns:

During the early stage of starvation (from CK to 2 weeks), many genes, specifically those involved in oxidative phosphorylation and thermogenesis pathways, were upregulated (Fig. 2E). Interestingly, pathways related to neurodegeneration were also upregulated. An overlap analysis of genes in these pathways revealed that most genes related to neurodegenerative pathways are also involved in oxidative phosphorylation and thermogenesis (Supplementary Fig. S2C). In the middle starvation phase (from 4 to 8 weeks), the number of differentially expressed genes (DEGs) was the lowest. Some downregulated genes were significantly enriched in the hippo signaling pathway and protein kinases (Fig. 2F). During the late starvation phase (from 14 to 19 weeks), pathways involved in protein transport and processing within the endoplasmic reticulum become particularly active (Fig. 2G). Interestingly, while the hippo pathway was upregulated at this stage, the key gene YAP (Hven08G09310) was present only in the downregulated hippo pathway during the middle-starvation period (Supplementary Table S7).

As PCA revealed a strong correlation between the expression profiles of the adipose tissue transcriptome and starvation tolerance duration in *H. venatoria*, we conducted a weighted gene coexpression network analysis (WGCNA) of the fat body transcriptome [51]. Clustering of the 18 fat body samples via WGCNA yielded a total of 9 modules, including the gray module (Supplementary Fig. S3A). Notably, the blue and brown modules exhibited significant correlations with the entire starvation process (Supplementary Fig. S3C, D). These 2 modules are hypothesized to play a dominant role in starvation tolerance. In the blue module, the majority of genes presented increased expression with prolonged starvation duration (Supplementary Fig. S4A), whereas a subset of genes was downregulated. Conversely, most genes in the brown module presented the opposite trend (Supplementary Fig. S4B). Functional enrichment analysis of the genes in these 2 modules revealed that, in addition to the previously mentioned AMPK signaling pathway, insulin resistance, and the adipocytokine signaling pathway, which are active in the later stages, the citrate cycle and peroxisome proliferator-activated receptor (PPAR) signaling pathway also exhibited heightened activity during the late stages of starvation.

### Final starvation stage in *H. venatoria*

The PCA results from both the fat body and whole-body transcriptomes indicated that the *H. venatoria* transcriptome at 19 weeks of starvation was markedly distinct from that at other stages (Fig. 2A, B). Compared with that in the other periods, the expression in the fat body and whole body was significantly correlated at 19 weeks (Fig. 2C and Supplementary Fig. S2B). Consequently, we conducted a differential analysis of the transcriptome at 19 weeks. Differential analysis of fat body tissue at 19 weeks revealed 612 upregulated genes and 647 downregulated genes (Supplementary Fig. S7A and Supplementary Table S5). The functional enrichment results revealed that only transporters and autophagy were significantly upregulated at 19 weeks, whereas energy-consuming pathways such as DNA replication and the cell cycle were essentially inactive (Supplementary Fig. S7B and Supplementary Table S5).

WGCNA of the fat body revealed that the lysosomal pathway is enriched in multiple modules in *H. venatoria*, indicating that the expression of different functional lysosomes during starvation in *H. venatoria* is also distinct. Specifically, the 6 genes in the blue module and the 11 genes in the brown module presented similar expression patterns during starvation, essentially showing continuous downregulation (Supplementary Fig. S4A, B, D, E), and the functional annotation results for these 17 genes indicated that they encoded mainly glycosidases, lipases, and proteases in the lysosome (Supplementary Table S3).

Interestingly, 24 genes of the lysosomal pathway in the turquoise module exhibited a sharp increase in expression at 14 weeks (Supplementary Fig. S4E, F and Supplementary Table S2). Compared with the aforementioned 17 genes, this group included more proteases. More importantly, 3 genes related to sulfatases were identified (Supplementary Table S3). The primary function of lysosomal sulfatases is to degrade sulfated glycosaminoglycans and glycolipids.

**Figure 3:**
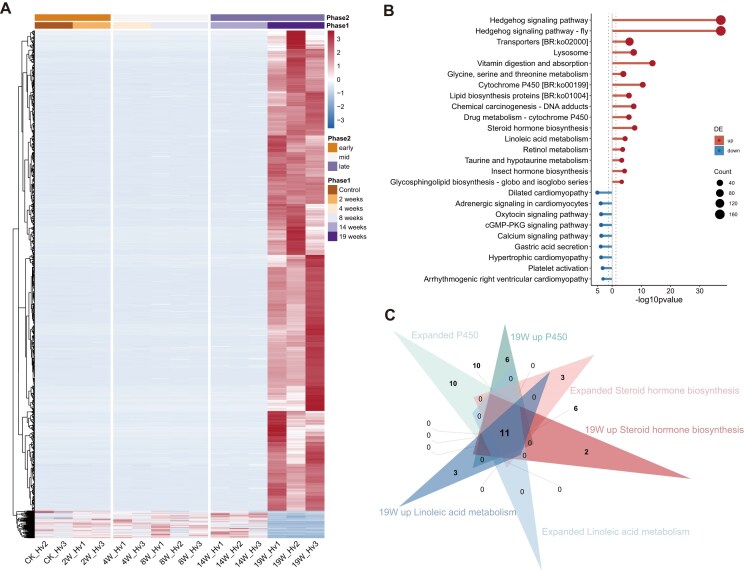
Whole-body transcriptome analysis of *Heteropoda venatoria* during a 19-week starvation period. (A, B) Heatmap and KEGG pathway enrichment analysis of DEGs in the whole-body transcriptome of *H. venatoria* at the 19-week starvation period. (C) Overlap of genes enriched in the cytochrome P450, steroid hormone biosynthesis, and linoleic acid metabolism pathways within the expanded gene families of *H. venatoria* and the same pathways identified at 19 weeks among upregulated genes of the whole-body transcriptome related to starvation resistance.

In contrast, the whole-body transcriptome at 19 weeks showed a substantial increase in upregulated genes, which was significantly greater than that in any other period (Fig. 3A). The functional enrichment results revealed that, in addition to the upregulation of pathways such as transporters and lysosome pathways, pathways such as cytochrome P450 [BR:ko00199], steroid hormone biosynthesis, and linoleic acid metabolism pathways also exhibited significant upregulation (Fig. 3B). These pathways have undergone notable gene family expansion in *H. venatoria* and are all related to lipid metabolism.

An overlap analysis of the genes enriched in these 3 expanded pathways and the genes upregulated at 19 weeks revealed 11 shared genes (Fig. 3C). The functional annotations of these 11 genes revealed that they are all associated with P450 (cytochrome P450), and interestingly, all are located on Chr4. We hypothesize that P450 genes play crucial roles in the starvation response of *H. venatoria*. Consequently, we conducted an identification analysis of P450 genes in *H. venatoria* and 11 other spider species.

### Changes in the expression of genes related to the lysosome, autophagy, and apoptosis pathways across various stages of starvation resistance

A study of the silkworm (*Bombyx mori*) revealed that prolonged starvation promotes both autophagy and apoptosis [52], with autophagy requiring degradation within lysosomes [53]. Given the large number of genes involved in the lysosome, autophagy, and apoptosis pathways but the relatively limited number of significantly enriched genes identified via differential expression analyses across various starvation stages, we aimed to more clearly observe the expression dynamics of all the genes annotated to these 3 pathways during starvation in *H. venatoria*. To achieve this goal, we conducted an expression trend analysis of all genes associated with these pathways at various stages of starvation. The results revealed 3 major expression trends for these pathways in the fat body: (i) expression levels were relatively low in the early and middle stages, peaked at 14 weeks, and then sharply declined at 19 weeks (Supplementary Fig. S9A, C, D, I); (ii) expression levels gradually decreased as starvation progressed (Supplementary Fig. S9E, H); and (iii) expression levels gradually increased as starvation progressed (Supplementary Fig. S9B). In the whole body, the expression trends for these 3 pathways were much more distinct: most genes presented low expression levels during the early and middle stages of starvation, followed by a sharp increase beginning at 14 weeks, or a gradual increase starting from the middle stage and persisting through the late stage of starvation (Supplementary Fig. S10A, C, D, E, H, I).

### P450 genes in 12 spiders

**Figure 4:**
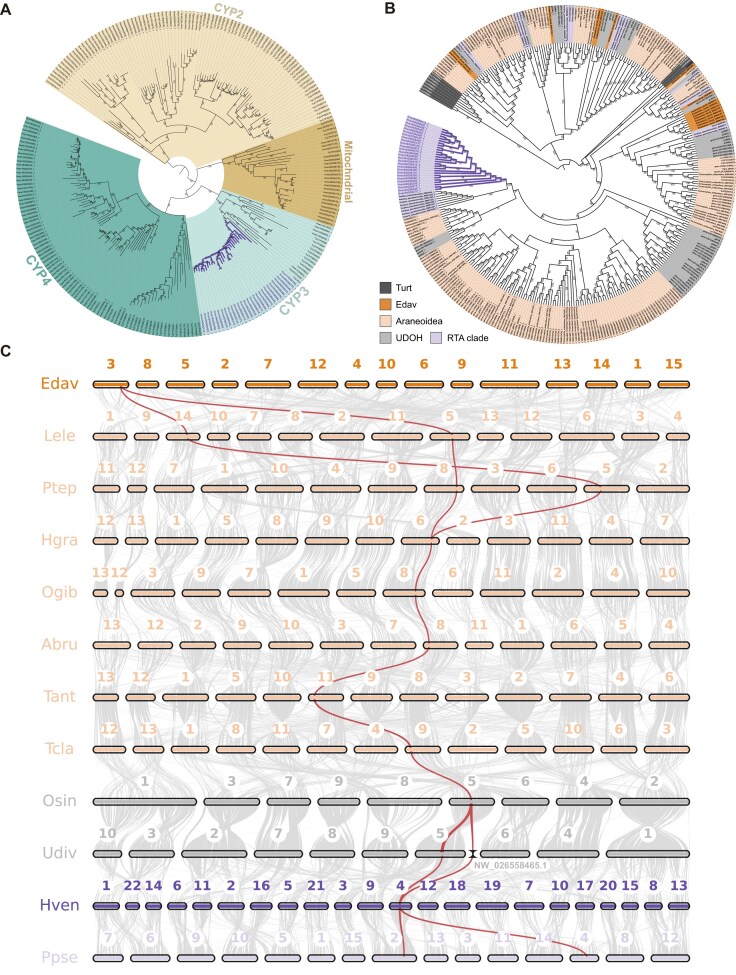
Evolutionary analysis of cytochrome P450 genes. (A) Phylogenetic tree of P450s in *Heteropoda venatoria* and *Tetranychus urticae*. (B) Phylogenetic tree of the CYP3 clan genes in 12 spider species and *T. urticae*. (C) Collinearity relationships of a subset of CYP3 clan genes in 12 spider species. The gray bands represent the connectivity between chromosome karyotypes and syntenic blocks, whereas the red lines indicate the collinearity of CYP3 genes. The numbers represent chromosome numbers, and NW_026,558,465.1 refers to a contig that has not been anchored to a chromosome.

The identification of P450 genes across 12 spider species revealed a total of 1,108 P450 genes encoding 1,270 P450 proteins. Among these, *H. venatoria* has the greatest number of P450 genes, totaling 141, whereas *Pardosa pseudoannulata*, which is also part of the RTA clade, has only 82 P450 genes. Phylogenetic tree construction revealed that spider P450 genes can be classified into the CYP2 clan, the CYP3 clan, the CYP4 clan and the mitochondrial clan (Fig. 4A and Supplementary Fig. S11). Notably, all 11 genes from the 3 enriched pathways mentioned earlier belong to the CYP3 clan. The phylogenetic tree for the CYP3 clan across these spiders revealed that 25 CYP3 clan genes from *H. venatoria* (representing 27 proteins) are located within a region of less than 2.5 Mb on Chr4 and cluster on the same branch (Fig. 4B). This finding also indicates significant expansion of the CYP3 subfamily in *H. venatoria*. These gene expansions likely reflect enhanced environmental adaptability in spiders, potentially influencing their metabolic capabilities, predation strategies, or ecological adaptations. We therefore conducted a synteny analysis of the proteins on this branch and the protein Ectatosticta_davidi_00,009,731_1 from *Ectatosticta davidi*, which is the closest relative to this branch.

Synteny analysis revealed that *H. venatoria* has an increased number of chromosomes due to extensive chromosomal fragmentation. In *Uloborus diversus*, members (gene-LOC129223072_1 and gene-LOC129233267_1) of this subfamily are located on Chr5 and have an unanchored scaffold. After chromosomal fragmentation, the main fragments of *U. diversus* Chr5 corresponded to Chr3 and Chr4 in *H. venatoria*, and significant gene duplication occurred on Chr4, leading to the expansion of the CYP3 subfamily (Fig. 4C).

Research has shown that a subfamily within CYP3, specifically CYP3A, can inhibit the metabolic rate of glucose in female mice, leading to an increase in fat [54]. As most genes of the CYP3 subfamily in *H. venatoria* are expressed in the fat body during various stages of starvation, we speculate that the numerous copies of the CYP3 subfamily genes maintain relatively low energy metabolism. This adaptation may allow *H. venatoria* to survive for extended periods without feeding. To further investigate the CYP3 subfamily in *H. venatoria*, we conducted additional sequence analyses.

### Conserved domains in P450s

Insect P450s are known to contain 5 conserved motifs: the helix C motif (WxxxR), the helix I motif (GxE/DTT/S), the helix K motif (ExLR), the PERF motif (PxxFxPE/DRE), and the heme-binding motif (PFxxGxRxCxG/A) [55, 56].

**Figure 5:**
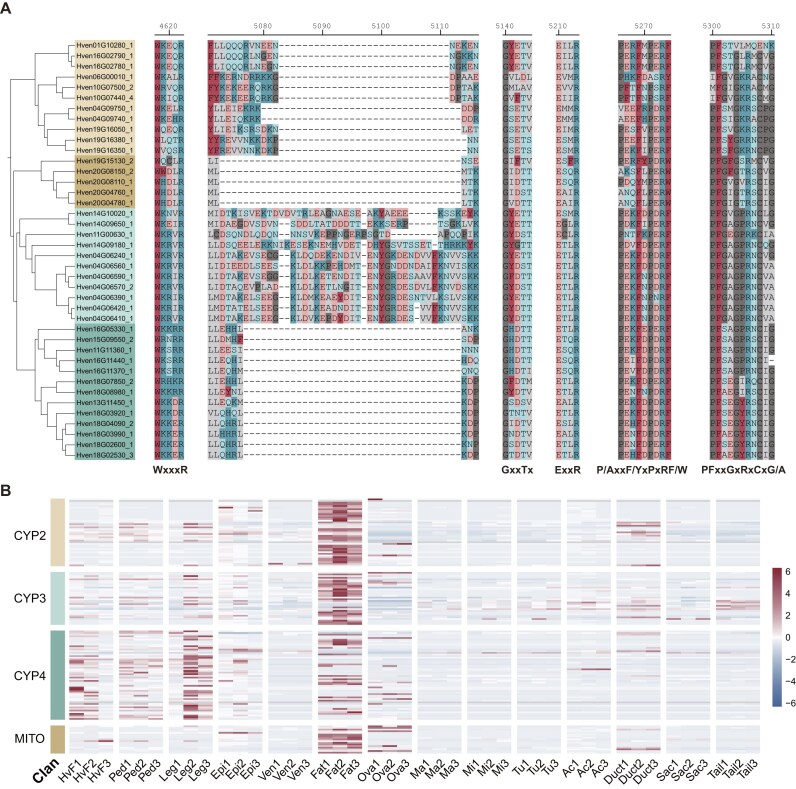
Analysis of conserved motifs in P450 genes and their expression across various tissues in *Heteropoda venatoria*. (A) The 5 common conserved motifs of the partial P450 genes and a specific sequence of the CYP3 genes in *H. venatoria*. (B) P450 gene expression in fat body (Fat) and other tissues (whole body of adult females [HvF], pedipalps [Ped], legs [Leg], epidermis [Epi], venom glands [Ven], ovaries [Ova], major ampullate glands [Ma], minor ampullate glands [Mi], tubuliform glands [Tu], aciniform glands [Ac], ducts of major ampullate glands [Duct], sacs of major ampullate glands [Sac], and tails of major ampullate glands [Tail]) in *H. venatoria*.

In *H. venatoria*, these 5 motifs are also found, but some of the amino acids within these motifs differ from those in insects. Therefore, we renamed P450 motifs in *H. venatoria* based on their characteristics: WxxxR, GxxTx, ExxR, P/AxxF/YxPxRF/W, and PFxxGxRxCxG/A (Fig. 5A and Supplementary Figs. S14, S15). Compared with the conserved motifs in insects, the motifs in spiders exhibit greater variability at many positions. Additionally, the syntenic relationships among spider genomes reveal extensive chromosomal breakage and fusion events (Fig. 4C), which undoubtedly increase the number of genomic polymorphisms in spiders; this likely contributes to the reduced number of conserved sites in spider P450 genes.

### High expression of P450 genes in the fat body

To further investigate the function of P450 genes, we analyzed the expression profiles of 4 P450 clans in various tissues of *H. venatoria*. The heatmap indicates that the CYP2, CYP3, and mitochondrial genes are predominantly expressed in the fat body, whereas the CYP4 genes are expressed not only in the fat body but also at significant levels in the pedipalps and legs (Fig. 5B). In comparison, in *P. pseudoannulata*, which belongs to the same RTA branch, the CYP2 and CYP3 genes are also highly expressed in the fat body, but the mitochondrial genes are not significantly expressed in the fat body [57]. Since transcriptomic data for the fat body of *Trichonephila clavata* are available, we also examined the expression of P450 genes in the fat body and other tissues of *T. clavata*. The analysis revealed that the CYP2, CYP3, and some CYP4 genes are highly expressed in the fat body in this species, whereas mitochondrial genes are predominantly expressed in the ovary (Supplementary Fig. S13).

## Discussion

In summary, our study is the first to systematically analyze gene expression differences in *H. venatoria* during various stages of starvation resistance, revealing metabolic pathways and signaling pathways associated with starvation tolerance. Through comparative analysis of the whole-body transcriptome and fat body transcriptome, we found that changes in the fat body transcriptome strongly correlated with starvation duration, suggesting that the fat body may play a crucial role in the starvation response of *H. venatoria*. In the early stages of starvation resistance, the upregulation of oxidative phosphorylation and thermogenic pathways indicates adequate functionality. Interestingly, we observed a significant downregulation of the key gene in the Hippo pathway, YAP, during the middle stage of starvation resistance. YAP has been found to be important for fat energy storage and expenditure [58]. Research on the Hippo signaling pathway and the regulation of cellular metabolism is increasing [58–60], leading us to hypothesize that this pathway, particularly the YAP gene, is vital in the starvation resistance process of *H. venatoria*. In the late stages of starvation resistance, the body faces significant energy supply pressure due to the substantial reduction in fat content. At this point, the upregulation of the AMPK pathway in the fat body aims to promote the oxidation of the remaining fatty acids [61].

Although the fat body of *H. venatoria* provides ample energy reserves, it must maintain a relatively low metabolic rate to slow energy consumption, allowing a starvation period of nearly 5 months. Interestingly, we found that P450 families play an important role in the fat body, with most P450 genes showing higher expression in this tissue than in other tissues. This pattern was observed not only in *H. venatoria* and *T. clavata* (Fig. 5B and Supplementary Fig. S13) but also in *P. pseudoannulata* [57]. During the starvation experiment, most P450 genes were expressed at various stages in the fat body (Supplementary Fig. S12A). However, the results from the whole-body transcriptome analysis were markedly different, with P450 genes showing higher expression at 19 weeks than at other times (Supplementary Fig. S12B). On the basis of existing research showing that some P450 families can reduce metabolic rates or participate in the regulation of lipid metabolism [54, 62–64], we hypothesize that during most starvation periods in *H. venatoria*, P450 genes are expressed primarily in the fat body to inhibit metabolic rates. As starvation progresses to the final stage (19 weeks), when energy reserves in the fat body are depleted, various *H. venatoria* tissues rely primarily on autophagy to function, with P450 gene expression triggered in most tissues to suppress metabolic rates.

The phylogenetic tree of spider P450 genes indicates that many spiders generate numerous copies within their genomes after P450 genes are acquired from their ancestors. *H. venatoria* shows the most significant expansion, with 25 clustered copies of the CYP3 family on Chr4, highlighting the crucial role this expansion plays in its survival in complex environments. This expansion significantly enhances the starvation tolerance of *H. venatoria*, making it more adept at coping with environmental changes and stresses. Moreover, the large number of gene copies increases redundancy, thereby protecting *H. venatoria* from the effects of harmful mutations. If 1 gene copy loses its function, other copies can still perform the necessary functions [65]. Synteny analysis revealed that these genes originated from *U. diversus* CYP3 clan genes. However, how these CYP3 clan genes in *H. venatoria* are duplicated and whether this duplication is related to complex chromosomal breakage and fusion phenomena in spiders require further research and exploration.

To increase the efficiency of fatty acid and amino acid transport, amino acid–related enzymes and transporters are upregulated synchronously during starvation in *H. venatoria*, promoting the transport of fatty acids and amino acids for energy; this may constitute an optimized energy utilization strategy, allowing the body to preserve crucial protein synthesis mechanisms in extreme environments. The gradual upregulation of the insulin-resistant and tricarboxylic acid (TCA) cycle indicates that fats and proteins become the primary energy sources in the middle to late stages of starvation. This metabolic reorganization reveals adaptive regulation in *H. venatoria* under extreme energy constraints.

The autophagy pathway plays a critical role in the late stage of starvation in *H. venatoria*. We observed that a significant number of genes in the autophagy, lysosome, and apoptosis pathways exhibited a sharp increase in expression in the whole-body transcriptome from 14 to 19 weeks (Supplementary Fig. S10A, D, I). Interestingly, many genes in these pathways presented a sharp decrease in expression during the same period in the fat body transcriptome (Supplementary Fig. S9A, C, D, I). We speculate that, as the primary energy-supplying organ in *H. venatoria*, the fat body was nearly fully depleted of its energy reserves through normal oxidative metabolism by 14 weeks. At this stage, the fat body could only rely on autophagy and apoptosis to process its remaining tissue. This explains why many genes in these pathways reached their peak expression levels at this time. However, energy production through these processes in the fat body alone was insufficient to sustain an organism’s overall energy demands. Consequently, autophagy and related responses in other tissues of *H. venatoria* became increasingly active and continued to intensify until 19 weeks. At this time, the fat body was essentially incapable of supplying energy, forcing most tissues in *H. venatoria* to depend on autophagy and apoptosis pathways for energy production, leading to the peak activity of these 3 pathways. Moreover, the upregulation of genes involved in sulfatase activity in the lysosomal pathway indicates that *H. venatoria* may begin to break down its internal connective tissues for energy under extreme starvation. These findings have significant ecological implications, as they provide insight into how *H. venatoria* and potentially other related species manage to survive in habitats with fluctuating food availability. Understanding the physiological and genetic mechanisms underlying starvation resistance in these spiders can inform broader ecological studies on predator–prey dynamics, resource allocation, and energy management within ecosystems [66]. Additionally, the ability to withstand prolonged periods of food scarcity might offer *H. venatoria* a competitive advantage in colonizing diverse and unpredictable environments, thereby influencing their distribution and ecological roles in different habitats. Understanding this physiological regulatory mechanism under severe energy constraints may be important for understanding the survival strategies and adaptive limits of arthropods.

## Methods

### Genome and Hi-C sequencing

We selected mature female *H. venatoria* whole tissue for library construction. Using PacBio HiFi sequencing for library preparation, we obtained high-quality full-length DNA for the entire genome. The DNA was fragmented using Megaruptor and then sorted by Sage ELF for 13–16 K fragments, followed by adapter ligation to obtain a SMRTbell library [25].

Next, we prepared the Hi-C library [67]. First, formaldehyde was used to fix DNA–protein or protein–protein complexes that were naturally cross-linked or spatially close within *H. venatoria* cells. Chromatin was subsequently digested and separated using the restriction enzyme DpnII, with end repair and biotin labeling of the fragment ends. DNA ligase was used to connect the ends, forming a circular chimeric molecule. These circular molecules were purified and then fragmented into DNA fragments. Finally, the biotin-labeled target DNA fragments were captured using a biotin precipitation technique, and DNA fragments of appropriate size were selected to establish the Hi-C library, which was then sequenced using the DNBseq platform for paired-end sequencing.

### Transcriptome sample processing

We selected mature female adult *H. venatoria* for this study. Preliminary tests of the starvation tolerance cycle revealed that the starvation period of *H. venatoria* was approximately 18 to 20 weeks. Therefore, the samples were divided into 6 groups according to the starvation stage: just after feeding (CK), 2 weeks after feeding (2 W), 4 weeks after feeding (4 W), 8 weeks after feeding (8 W), 14 weeks after feeding (14 W), and 18–20 weeks after feeding (determined by the spider’s condition). The environmental temperature was set at 22 ± 2°C during the day (9:00–19:00) and 16 ± 2°C at night (19:00 to next day at 9:00), with a humidity of 70 ± 10% and a natural light cycle. Both the adipose tissue and the entire spider were sampled from each group for transcriptome sequencing. Before the formal starvation experiment, preliminary processing was necessary to ensure that there were enough samples that could feed within the same time frame. After the samples were obtained, unrestricted feeding was allowed during the first week, and feeding was stopped in the second week for 1 week to ensure that most of the spiders were in a state of hunger. On the first day of the third week, formal feeding commenced, and samples were selected for subsequent experiments within 3 hours. The spiders were divided into 6 groups, with 8 spiders in each group (including 2 as backups). In the first group (CK), the adipose tissue and the entire spider were sampled from 3 samples each 3 hours after feeding; in the second group, the tissues were sampled after 2 weeks of starvation, with 3 replicates totaling 6 samples and so on. Sampling was conducted when the last group began to show spider death, and ultimately the spiders died during the 19th week of starvation; thus, the last group was set as 19 weeks of starvation. In total, we obtained 6 groups of fat body samples and 6 groups of whole-body samples, totaling 36 samples.

### Transcriptome sequencing

A certain amount of RNA sample was taken and used to obtain mRNA from total RNA using oligo(dT). The mRNA was then fragmented, and random primers were subsequently used for cDNA synthesis. During the synthesis of the second strand of cDNA, dUTP was used instead of dTTP. The double-stranded cDNA was subjected to end repair, “A” addition, and adapter ligation. The enzyme UDG was used to digest the U-tagged second-strand template, followed by PCR and PCR product recovery. The library quality was assessed, and upon qualification, the product was circularized. The circular DNA molecules were subjected to rolling circle replication to form DNA nanoballs (DNBs) [68], which were then sequenced on the DNBSEQ platform.

### Genome assembly

Hifiasm v0.16.1 (RRID:SCR_021069) software was used with default parameters to perform an initial assembly of HiFi reads [26], resulting in contigs. The raw Hi-C reads were subsequently filtered using Hic-pro v3.1.0 (RRID:SCR_017643) [69], and the filtered Hi-C reads were subsequently analyzed with Juicer v1.6 to obtain a Hi-C interaction matrix [70]. 3D-DNA v201008 was then employed to scaffold the contigs, yielding an initial pseudo-chromosomal genome [71]. Finally, manual corrections were applied using Juicerbox v1.11.08 to produce the final chromosomal genome.

### Repeat annotation

Repeat annotation consists of 2 parts, namely, utilizing an existing repeat library for repeat identification and constructing a repeat library from the genome itself for repeat identification, with the results from both parts being combined. RepeatMasker v4.1.2 (RRID:SCR_012954) [72] was used with the known repeat library Repbase v20181026 (RRID:SCR_021169) [73] for preliminary repeat identification. The construction of a self-derived repeat library was subsequently carried out using MITE Tracker v2.7.1 (RRID:SCR_017030) with default parameters to construct the mite library [74], followed by LTR analysis using ltrharvest v1.6.2 (RRID:SCR_018970) [75] and LTR_FINDER_parallel v1.1 (RRID:SCR_018969) [76], and the LTR library was integrated using LTR_retriever v2.9.5 (RRID:SCR_017623) [77]. RepeatModeler v2.0.2 (RRID:SCR_015027) was used for repeat analysis to obtain the repeat library [78]. The repeat libraries obtained from these tools were then integrated, and redundant sequences were removed using vsearch v2.23.0 (RRID:SCR_024494) to produce the final repeat library [79]. Repeat identification was then performed using the repeatmasker parameter (-lib) specifying this library, and the results from both identification processes were consolidated using the ProcessRepeats script included with RepeatMasker.

### Gene structure annotation

Initially, *de novo* gene structure prediction was performed using Augustus v3.4.0 (RRID:SCR_008417) [80] and SNAP (RRID:SCR_007936) [81]. The assembled transcripts were subsequently obtained using HISAT2 v2.2.1 (RRID:SCR_015530) [82] and StringTie v2.2.1 (RRID:SCR_016323) [83]. Transcriptomic evidence annotation was then conducted with PASA v2.5.2 [84]. Homology annotation was carried out using exonerate v2.4.0 (RRID:SCR_016088) [85] and GeMoMa v1.9 (RRID:SCR_017646) [86] with proteins from closely related species. Finally, the results from these 3 annotation methods were integrated using MAKER v3.01.04 (RRID:SCR_005309) [87] to produce the final annotation.

### GO functional annotation

Using BLASTP v2.12.0 (RRID:SCR_001010) (e value ≤ 1e-5) [88], spider protein sequences were aligned against homologous protein sequences in the UniProt (RRID:SCR_002380) Knowledgebase (UniProtKB) database [89], with the best alignments selected on the basis of bit score values. The GO annotations for the species were determined based on the annotation information of the similar proteins. ID mapping was performed using the IDmapping file, where the first column represents the UniProtKB ID and the eighth column contains the GO annotations [90].

### KEGG annotation

KEGG annotation of genes was performed using KofamScan v1.3.0 [91], with the output format set to mapper and an e-value threshold of 1e-5.

### Genome size and repeat correlation analysis

In addition to the *H. venatoria* genome, the genome sequences of a scorpion and 12 other spider species were collected and subjected to repeat analysis. The correlation between the proportions of different types of repeat sequences and genome size across the 14 species was calculated using the cor function from the R package stats v4.2.2 [92], with the analysis method set to Pearson. A correlation heatmap was generated using the corrplot v0.92 package [93].

### Phylogenetic tree construction

To reconstruct the evolutionary history of *H. venatoria*, a dataset comprising 12 Araneae species and a Scorpiones outgroup (*Centruroides sculpturatus*) was utilized for maximum likelihood tree construction. First, we ran OrthoFinder v2.5.4 [94] to infer orthologs using BLASTP v2.12.0 [88] with a *P* value threshold of <1e-5, resulting in the identification of 1,764 one-to-one orthologous sequences. Orthologs were aligned using MAFFT v7.520 (RRID:SCR_011811) [95] with the accurate option (L-INS-i) and trimmed using trimAl v1.4.rev15 [96] with the automated1 parameter. The trimmed alignments were then concatenated to serve as input for IQ-TREE v2.2.2.7 (RRID:SCR_017254) [97], using ModelFinder Plus (MFP) mode and 1,000 bootstrap replicates.

### Gene family expansion and contraction analysis

We used CAFE5 v5.1.0 to investigate gene family expansion and contraction across selected species [98]. An ultrametric species tree was obtained using the MCMCTREE program in PAML v4.10.7 (RRID:SCR_014932) [99]. The calibration of the divergence time for species was derived from Magalhaes, Timetree, and Paleobiodb with Scorpiones stem (418–423 million years ago [Mya]); the split between *E. davidi* and 11 other spiders (242–299 Mya); and the split between *U. diversus* and *H. venatoria* (173–240 Mya) [100, 101].

The gene family results were acquired from OrthoFinder, with only those families containing no more than 100 gene copies retained for further analysis. In CAFE5, the base model and unspecified Poisson distribution were used to conduct calculations. The significantly expanded families (*P* < 0.05) in *H. venatoria* were analyzed for functional enrichment using the R package clusterProfiler v4.10.1 (RRID:SCR_016884) [102].

### Transcriptome analysis

PCA was conducted using the R package FactoMineR v2.10 (RRID:SCR_014602) [103], and differential transcriptomic analysis was performed with the DESeq2 v1.42.1 package [104]. For the differential analysis of fat body stages (early, middle, and late), taking the early stage as an example, DEGs were identified for both the control (CK) and 2-week samples relative to the middle- and late-stage samples. The logFoldChange threshold was set at 1, and a *P* value of 0.05 was considered to indicate statistical significance. The intersection of the 2 sets of DEGs was taken as the early-stage DEGs. The middle and late stages were analyzed similarly.

DEGs at the 19-week stage in the fat body and whole-body tissues were analyzed relative to those at other stages. Using the fat body as an example, DEGs were calculated separately for 19 weeks relative to the CK, 2-week, 4-week, 8-week, and 14-week periods. The intersection of these 5 sets of DEGs was considered to represent the DEGs at 19 weeks in the fat body. The logFoldChange threshold was set at 0.5, and a *P* value of 0.05 was considered significant for fat body differential analysis. For whole-body analysis, the logFoldChange threshold was also set at 0.5, with a *P* value of 0.05 considered indicative of statistical significance.

WGCNA was performed on the fat body transcriptome via the R package WGCNA v1.72.5 [51], with the minimum module gene count set at 30 and the soft threshold power in fit indices set at 10. For functional enrichment analysis, KEGG pathway enrichment was performed using the clusterProfiler v4.10.1 package, with a corrected *P*-adjust value less than 0.05 considered indicative of statistical significance.

To observe the gene expression trends of the autophagy, lysosome, and apoptosis pathways at different stages of starvation resistance, we extracted the expression matrices of all genes annotated to these 3 pathways from the fat body and whole-body transcriptomes. We performed gene expression clustering based on the fuzzy c-means algorithm via the R package Mfuzz v2.62.0 (RRID:SCR_000523) [105]. Finally, we visualized the results using the mfuzz.plot2.

### Cytochrome P450 family analysis

Three approaches were utilized to identify putative CYP genes. First, we downloaded known CYP amino acid sequences of 5 arthropods, namely, *Apis mellifera, Bombyx mori, Drosophila melanogaster, Pogonomyrmex barbatus*, and *Tetranychus urticae*, from the Cytochrome P450 Homepage. We used the protein sequences of 12 Araneae species as queries to perform BLASTP v 2.12.0+ analysis against known CYPs, employing a threshold of *P* < 1e-30. Second, we used the hmmersearch program within HMMER v3.3.2 (RRID:SCR_005305) [106]. The P450 domain (PF00067) was searched against the candidate gene from the BLAST results, which required both full sequence and domain scores >100. Finally, we manually removed the sequences that lacked any 1 of the 5 conserved motifs of CYPs (WxxxR, Gx[ED]T[TS], ExLR, PxxFxP[ED]RF, PFxxGxRxCx[GA] in Insecta).

For CYP family phylogeny, sequences of Araneae species and *Tetranychus urticae* were aligned with MAFFT (L-INS-i) and trimmed with trimAl (gappyout method). The trimmed sequences were then input to IQTREE2 (MFP mode), and a CYP phylogenetic tree was constructed. All trees presented in our article were visualized using iTOL v6 (RRID:SCR_018174) [107].

### Synteny analysis

To assess the genomic synteny between *H. venatoria* and other species, we employed the JCVI v1.3.8 MCScan application for the calculations [108]. We have highlighted a specific clade within the CYP clan 3 members in the figure instead of a synteny block.

## Supplementary Material

giaf019_Supplemental_File

giaf019_GIGA-D-24-00314_Original_Submission

giaf019_GIGA-D-24-00314_Revision_1

giaf019_GIGA-D-24-00314_Revision_2

giaf019_GIGA-D-24-00314_Revision_3

giaf019_Response_to_Reviewer_Comments_Original_Submission

giaf019_Response_to_Reviewer_Comments_Revision_1

giaf019_Response_to_Reviewer_Comments_Revision_2

giaf019_Reviewer_1_Report_Original_Submission Hui Xiang, PH.D -- 9/17/2024

giaf019_Reviewer_1_Report_Revision_1 Hui Xiang, PH.D -- 12/26/2024

giaf019_Reviewer_2_Report_Original_Submission Sandra Correa-Garhwal -- 9/27/2024

## Data Availability

All high-throughput raw sequencing data, the genome assembly, and gene annotation data used in this project were deposited into the Genome Sequence Archive (GSA) of the National Genomics Data Center (NGDC) through BioProject PRJCA014503 and NCBI BioProject PRJNA1146965. All additional supporting data are available in the *GigaScience* repository, GigaDB [109].

## Additional Files

**Supplementary Fig. S1**. Image and relevant statistics of *Heteropoda venatoria*. (A) Image of a female *H. venatoria*. (B) Length statistics of the HiFi sequencing reads. (C) Results of the genome survey. (D) Significance statistics of the GC content in repetitive sequences, as well as the LTR and TcMar sequences, compared with nonrepetitive sequences in the genome of *H. venatoria*.

**Supplementary Fig. S2**. Transcriptomic analysis results of the fat body and whole body of *Heteropoda venatoria*. (A) Principal component analysis (PCA) results of the starvation resistance of all whole-body samples. (B) Correlation analysis of the transcriptomes of the fat body and whole body across various stages of starvation resistance. (C) Overlap of genes enriched in pathways related to thermogenesis, oxidative phosphorylation, and neurodegenerative diseases. (D) Soft thresholds of the fat body and whole body in WGCNA.

**Supplementary Fig. S3**. The results of WGCNA. (A) The modules clustered of WGCNA in fat body transcriptomes. (B) Correlations between modules and traits. (C, D) Scatterplot of gene significance (y-axis) vs. module membership (x-axis) in the blue and brown modules.

**Supplementary Fig. S4**. The expression and KEGG functional enrichment results of modules with WGCNA. (A–C) The expression of the blue, brown, and turquoise modules. (D–F) KEGG functional enrichment results of the blue, brown, and turquoise modules.

**Supplementary Fig. S5**. The analysis results of the blue module in WGCNA. (A) The expression trends of the genes enriched in the 4 pathways during the early, middle, and late periods of starvation resistance. (B) Network of transporter pathway-related genes enriched in the blue module. (C) Network of 4 energy metabolism-related pathway (AMPK, the TCA cycle, PPAR, and insulin resistance) genes enriched in the blue module.

**Supplementary Fig. S6**. The expression trends of pathways enriched with DEGs in the fat body transcriptome of *Heteropoda venatoria* during a 19-week starvation period.

**Supplementary Fig. S7**. Heatmap and KEGG pathway enrichment analysis of DEGs in the fat body transcriptome of *Heteropoda venatoria* after 19 weeks of starvation resistance.

**Supplementary Fig. S8**. The expression of all genes involved in the autophagy, lysosome, and apoptosis pathways across various stages of starvation resistance in *Heteropoda venatoria*. (A–C) Heatmap of autophagy pathway, lysosome pathway, and apoptosis pathway in the fat body transcriptome. (D–F) Heatmap of autophagy pathway, lysosome pathway, and apoptosis pathway in the whole-body transcriptome.

**Supplementary Fig. S9**. Trend analysis of the expression of all genes involved in the autophagy, lysosome, and apoptosis pathways in the fat body across various stages of starvation resistance in *Heteropoda venatoria*. (A–C) Trend analysis of the expression of the autophagy pathway, lysosome pathway, and apoptosis pathway, with 67, 139, and 61 genes, respectively, expressed in the fat body.

**Supplementary Fig. S10**. Trend analysis of the expression of all genes involved in the autophagy, lysosome, and apoptosis pathways in the whole body across various stages of starvation resistance in *Heteropoda venatoria*. (A–C) Trend analysis of the expression of the autophagy pathway, lysosome pathway, and apoptosis pathway, with 68, 141, and 61 genes, respectively, expressed in the whole body.

**Supplementary Fig. S11**. Phylogenetic tree of all P450 genes in 12 spiders and *Tetranychus urticae*.

**Supplementary Fig. S12**. The expression of P450 genes in the fat body and whole body across various stages of starvation resistance in *Heteropoda venatoria*.

**Supplementary Fig. S13**. The expression of P450 genes in various tissues of *Trichonephila clavata*.

**Supplementary Fig. S14**. The 5 common conserved motifs of all P450 genes and a specific sequence of CYP3 genes in *Heteropoda venatoria*.

**Supplementary Fig. S15**. The seqLogo of conserved motifs of P450 genes in spiders.

**Supplementary Table S1**. The functional enrichment results of expanded family genes.

**Supplementary Table S2**. The functional enrichment results of the blue, brown, and turquoise modules.

**Supplementary Table S3**. KEGG annotations of lysosome genes with blue, brown, and turquoise modules.

**Supplementary Table S4**. The list of expanded family genes.

**Supplementary Table S5**. DEGs and their functional enrichment results of the fat body and whole-body transcriptomes of *Heteropoda venatoria* at the 19-week starvation period.

**Supplementary Table S6**. GC contents of repeat region, nonrepeat region, LTRs, and TcMar elements in the *Heteropoda venatoria* genome.

**Supplementary Table S7**. The functional enrichment results of DEGs in the fat body transcriptome during the early, middle, and late stages of starvation resistance.

**Supplementary Table S8**. The list of genes annotated to the autophagy, lysosome, and apoptosis pathways.

## Abbreviations

BLAST: Basic Local Alignment Search Tool; BUSCO: Benchmarking Universal Single-Copy Orthologs; DEG: differentially expressed gene; DNB: DNA nanoball; GO: Gene Ontology; KEGG: Kyoto Encyclopedia of Genes and Genomes; Mya: million years ago; PCA: principal component analysis; WGCNA: weighted gene coexpression network analysis.
