# Supplementary material for: Genomic and transcriptomic analyses of Heteropoda venatoria reveal the expansion of P450 family for starvation resistance in spiders
Source: Gigascience. 2025 Mar 21;14:giaf019. doi: 10.1093/gigascience/giaf019 (PMC11927401; doi:10.1093/gigascience/giaf019)
Supplement: giaf019_Supplemental_File [file giaf019_supplemental_file.zip › FigureS3.pdf]

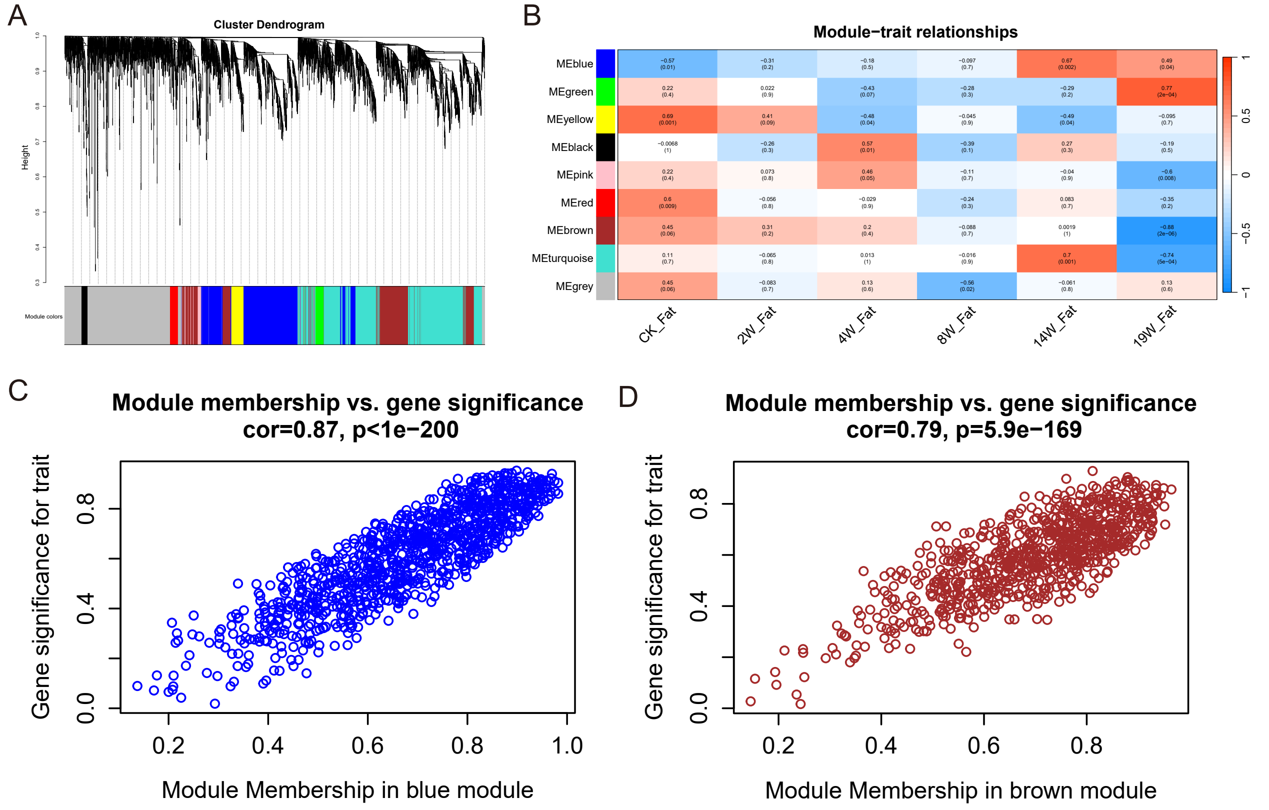

A
Cluster Dendrogram
Height
1.0
0.9
0.8
0.7
0.6
0.5
0.4
0.3
Module colors
B
Module−trait relationships
MEblue
MEgreen
MEyellow
MEblack
MEpink
MEred
MEbrown
MEturquoise
MEgrey
−0.57 (0.01)
−0.31 (0.2)
−0.18 (0.5)
−0.097 (0.7)
0.67 (0.002)
0.49 (0.04)
0.22 (0.4)
0.022 (0.9)
−0.43 (0.07)
−0.28 (0.3)
−0.29 (0.2)
0.77 (2e−04)
0.69 (0.001)
0.41 (0.09)
−0.48 (0.04)
−0.045 (0.9)
−0.49 (0.04)
−0.095 (0.7)
−0.0068 (1)
−0.26 (0.3)
0.57 (0.01)
−0.39 (0.1)
0.27 (0.3)
−0.19 (0.5)
0.22 (0.4)
0.073 (0.8)
0.46 (0.05)
−0.11 (0.7)
−0.04 (0.9)
−0.6 (0.008)
0.6 (0.009)
−0.056 (0.8)
−0.029 (0.9)
−0.24 (0.3)
0.083 (0.7)
−0.35 (0.2)
0.45 (0.06)
0.31 (0.2)
0.2 (0.4)
−0.088 (0.7)
0.0019 (1)
−0.88 (2e−06)
0.11 (0.7)
−0.065 (0.8)
0.013 (1)
−0.016 (0.9)
0.7 (0.001)
−0.74 (5e−04)
0.45 (0.06)
−0.083 (0.7)
0.13 (0.6)
−0.56 (0.02)
−0.061 (0.8)
0.13 (0.6)
CK_Fat
2W_Fat
4W_Fat
8W_Fat
14W_Fat
19W_Fat
1
0.5
0
−0.5
−1
C
Module membership vs. gene significance
cor=0.87, p<1e−200
Gene significance for trait
0.0
0.4
0.8
0.2
0.4
0.6
0.8
1.0
Module Membership in blue module
D
Module membership vs. gene significance
cor=0.79, p=5.9e−169
Gene significance for trait
0.0
0.4
0.8
0.2
0.4
0.6
0.8
Module Membership in brown module
