# Supplementary material for: Genomic and transcriptomic analyses of Heteropoda venatoria reveal the expansion of P450 family for starvation resistance in spiders
Source: Gigascience. 2025 Mar 21;14:giaf019. doi: 10.1093/gigascience/giaf019 (PMC11927401; doi:10.1093/gigascience/giaf019)
Supplement: giaf019_Supplemental_File [file giaf019_supplemental_file.zip › FigureS6_revision2.pdf]

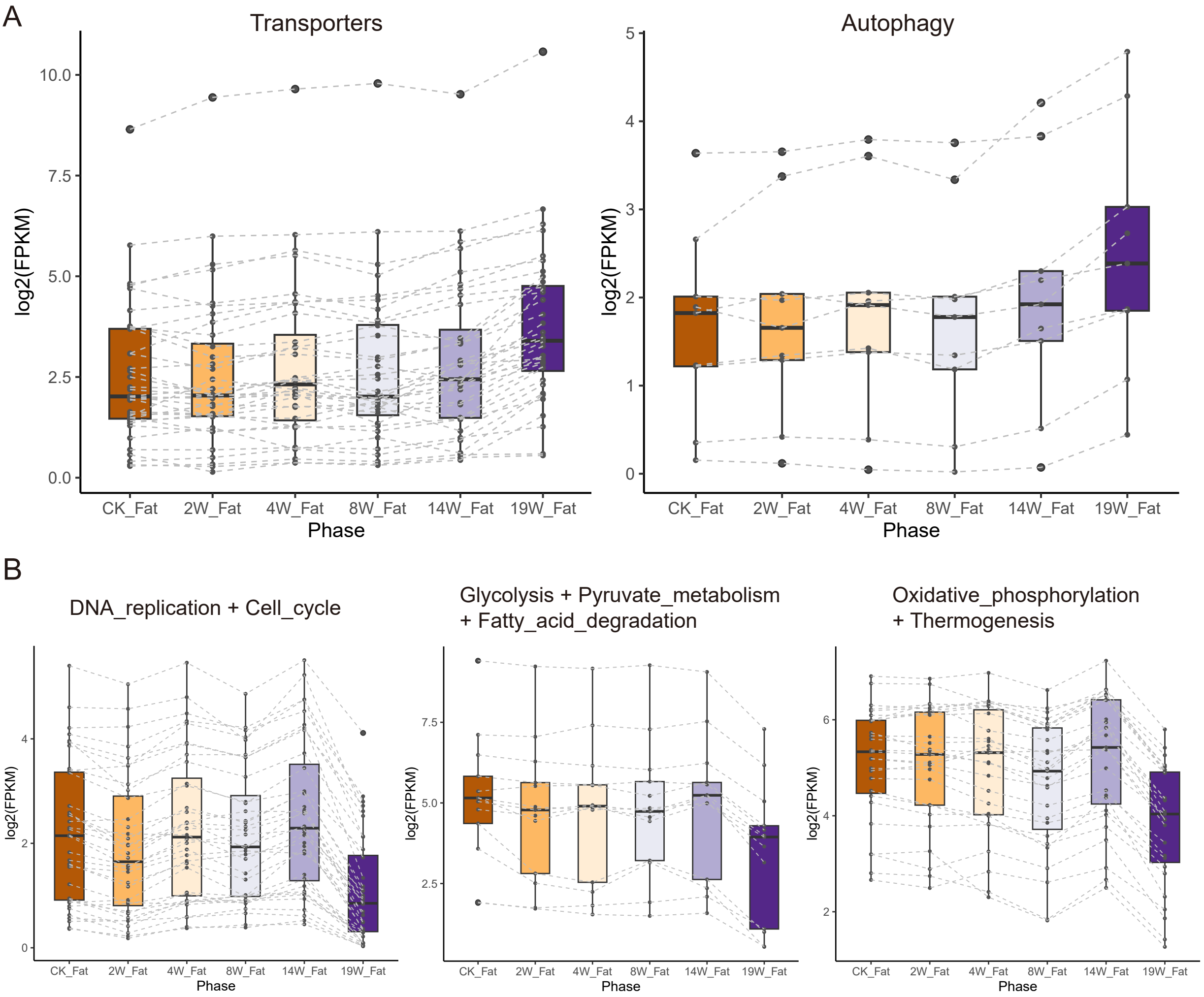

A
Transporters
Autophagy
log2(FPKM)
Phase
CK_Fat
2W_Fat
4W_Fat
8W_Fat
14W_Fat
19W_Fat
0.0
2.5
5.0
7.5
10.0
0
1
2
3
4
5
B
DNA_replication + Cell_cycle
Glycolysis + Pyruvate_metabolism
+ Fatty_acid_degradation
Oxidative_phosphorylation
+ Thermogenesis
0
2
4
2.5
5.0
7.5
6
