# Supplementary figures and images for: Genomic and transcriptomic analyses of Heteropoda venatoria reveal the expansion of P450 family for starvation resistance in spiders
Source: Gigascience. 2025 Mar 21;14:giaf019. doi: 10.1093/gigascience/giaf019 (PMC11927401; doi:10.1093/gigascience/giaf019)

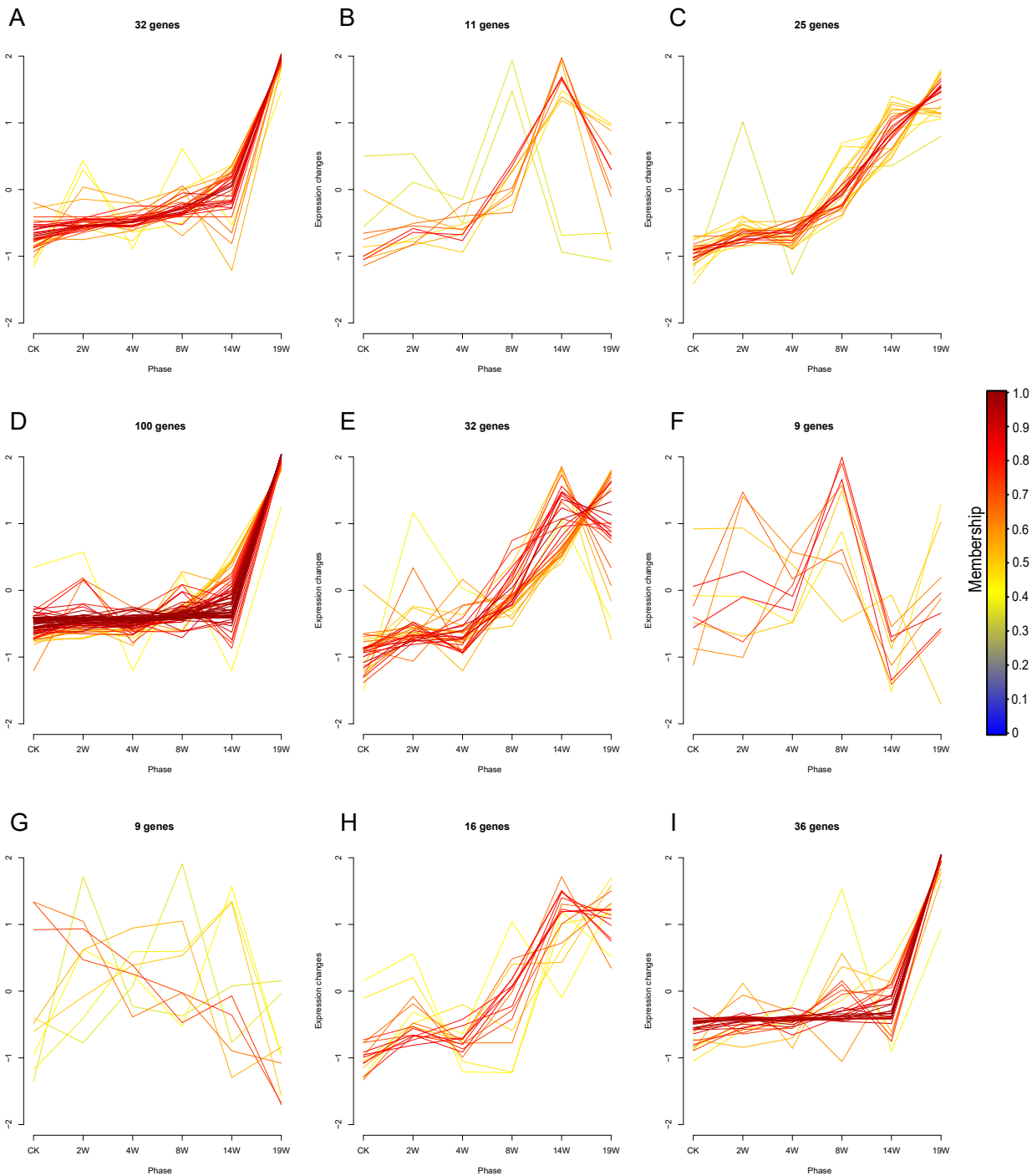

Supplement: giaf019_Supplemental_File [file giaf019_supplemental_file.zip › FigureS10_revision2.pdf]

Tree scale: 1

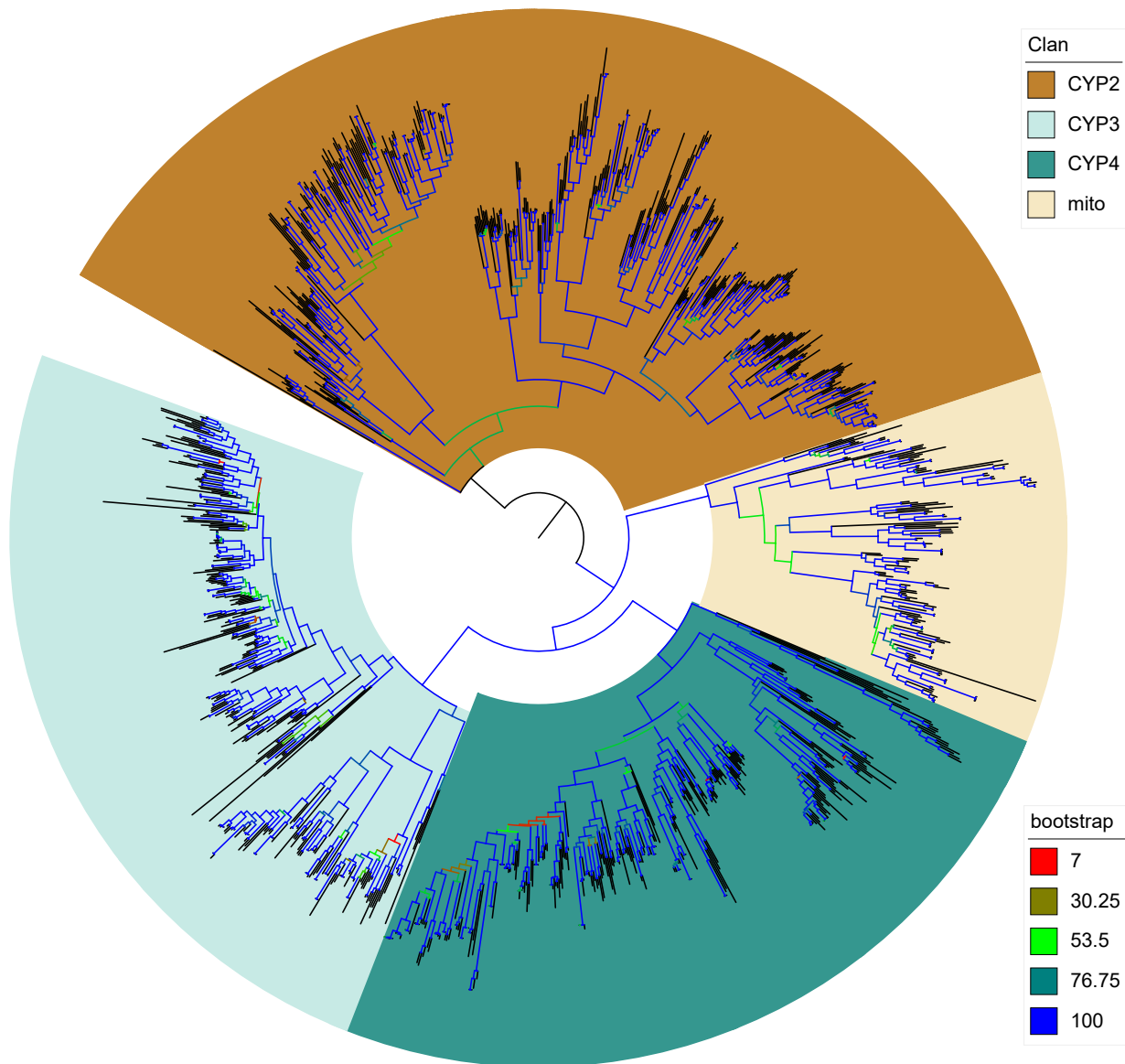

Supplement: giaf019_Supplemental_File [file giaf019_supplemental_file.zip › FigureS11_revision2.pdf]

A

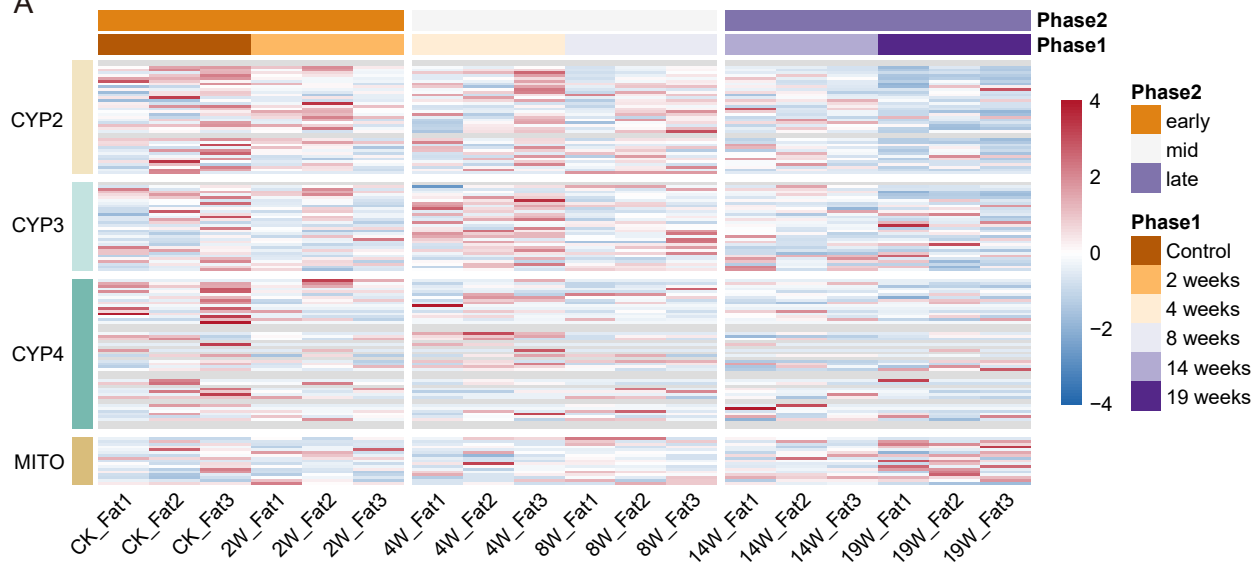

B

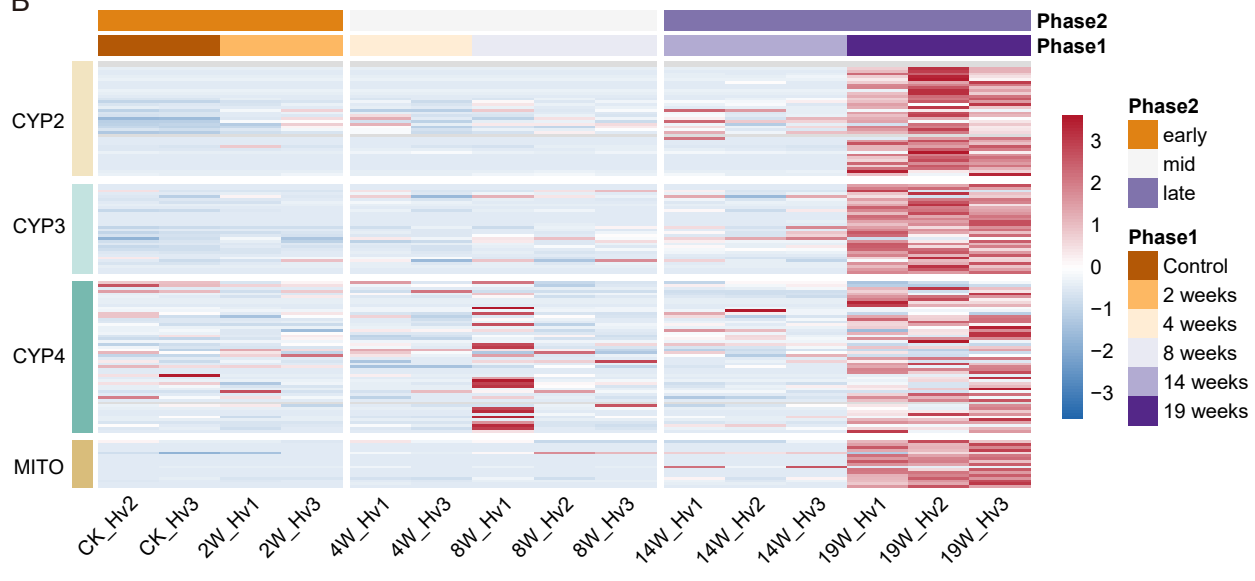

Supplement: giaf019_Supplemental_File [file giaf019_supplemental_file.zip › FigureS12_revision2.pdf]

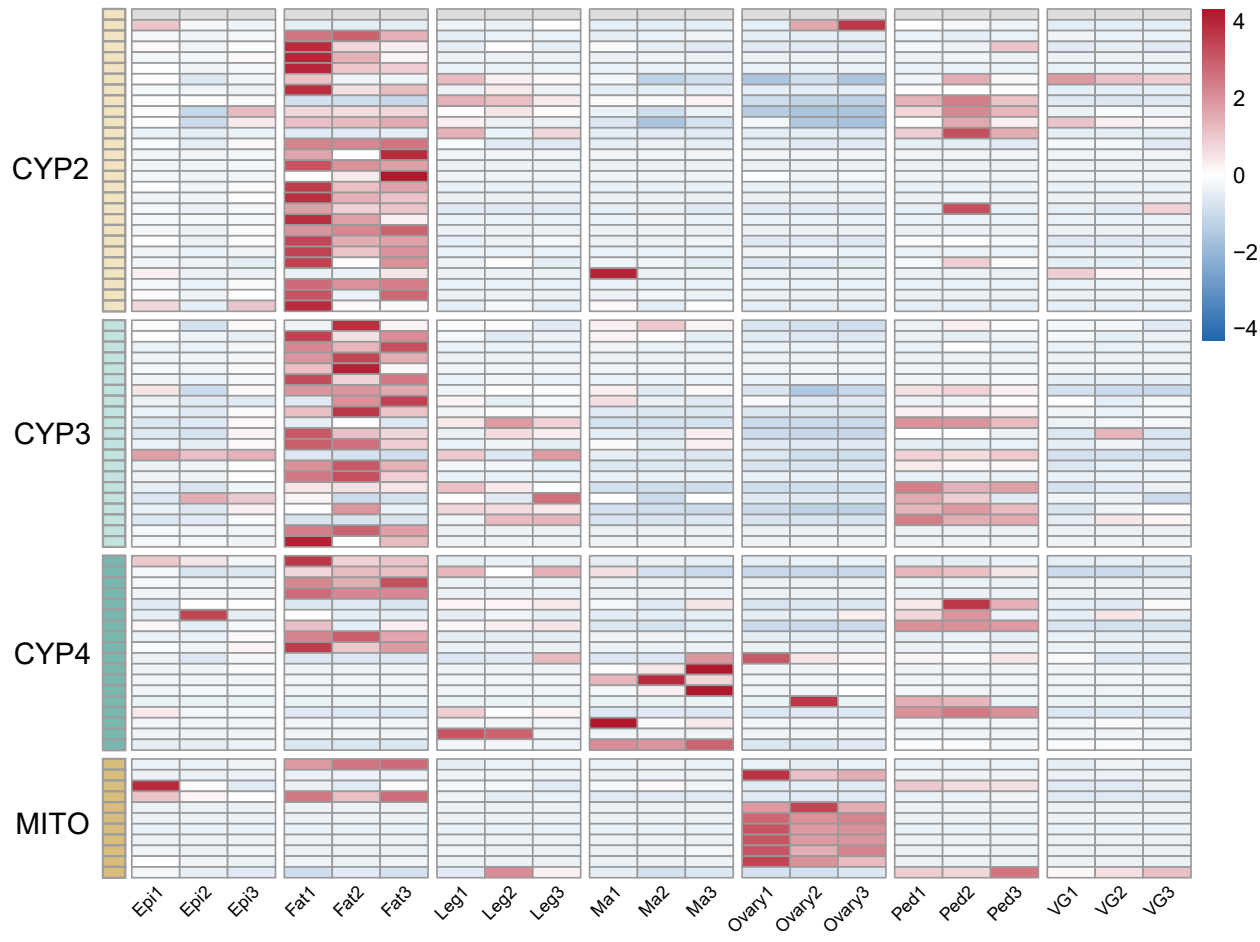

Supplement: giaf019_Supplemental_File [file giaf019_supplemental_file.zip › FigureS13_revision2.pdf]

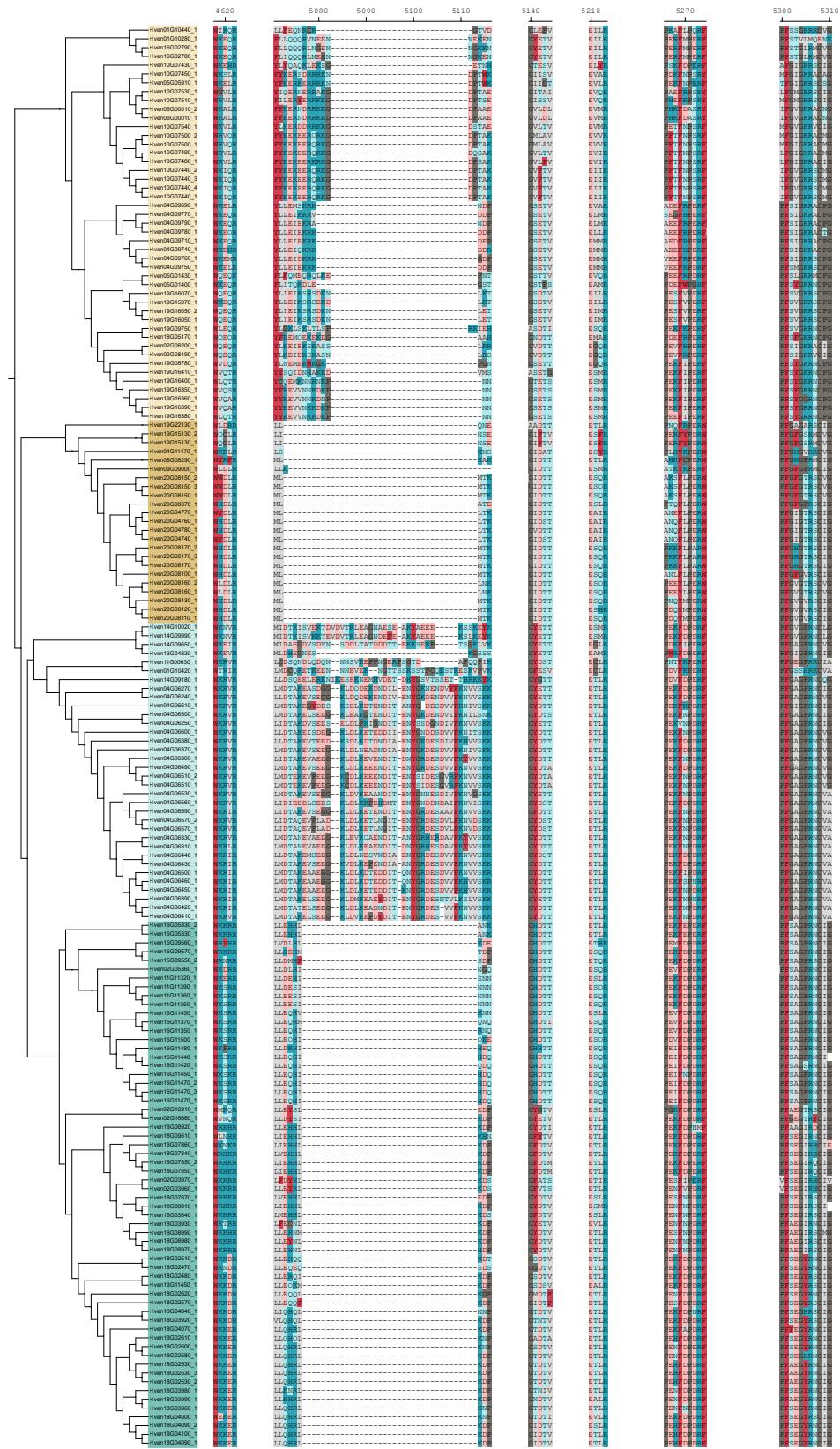

WxxxR

Gxxxx

ExxR

P/AxxF/y/xPxRf/W

PFxxGxRxCxG/A

Supplement: giaf019_Supplemental_File [file giaf019_supplemental_file.zip › FigureS14_revision2.pdf]

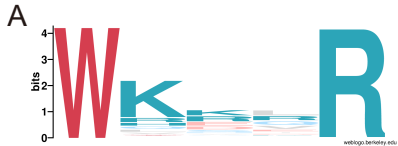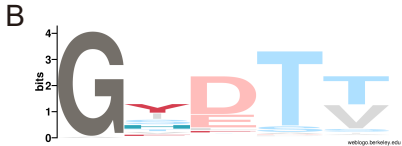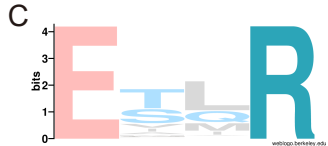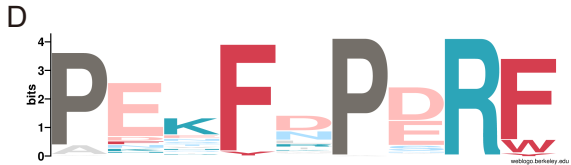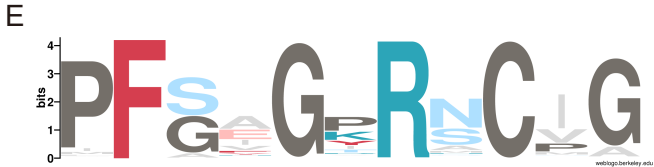

Supplement: giaf019_Supplemental_File [file giaf019_supplemental_file.zip › FigureS15_revision2.pdf]

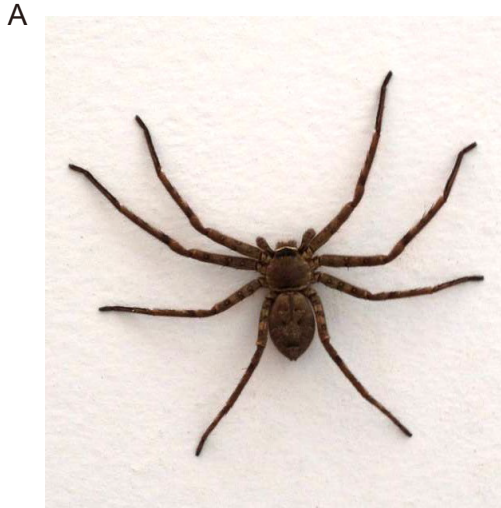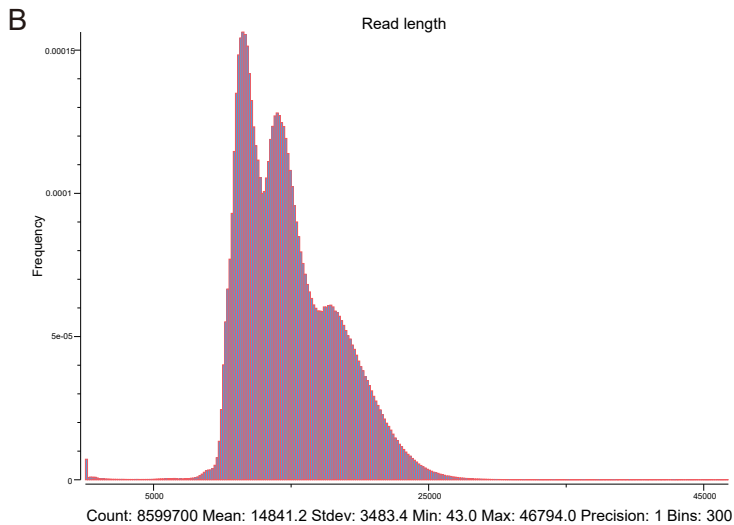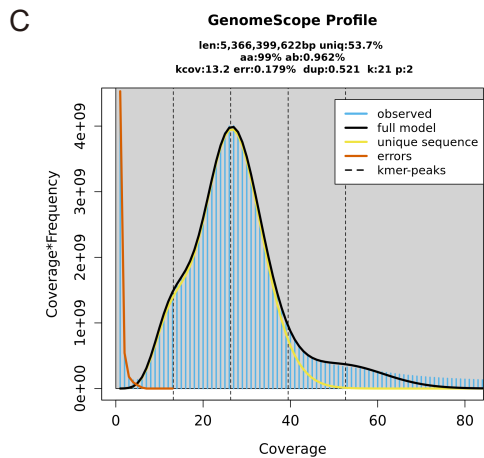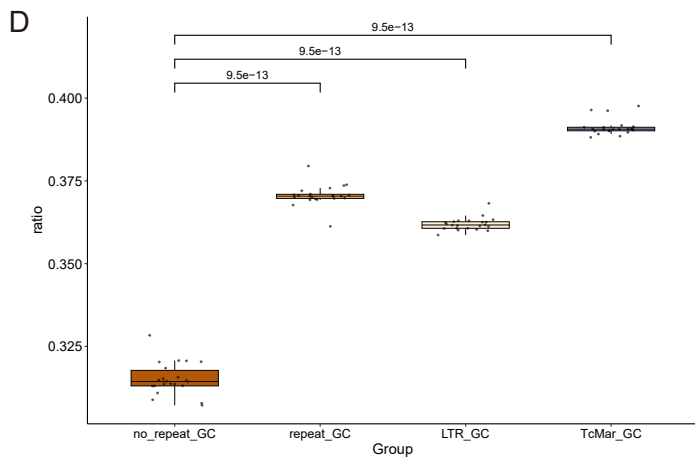

Supplement: giaf019_Supplemental_File [file giaf019_supplemental_file.zip › FigureS1_revision1.pdf]

A

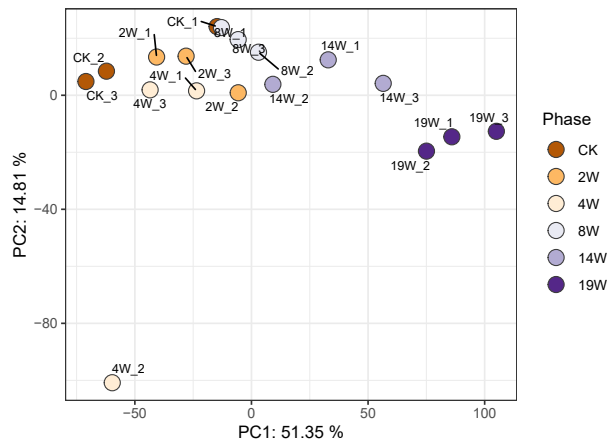

B

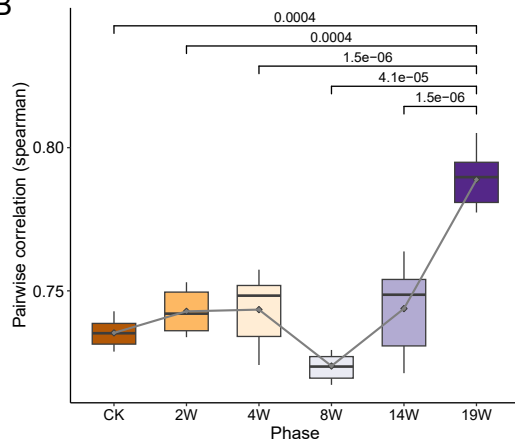

C

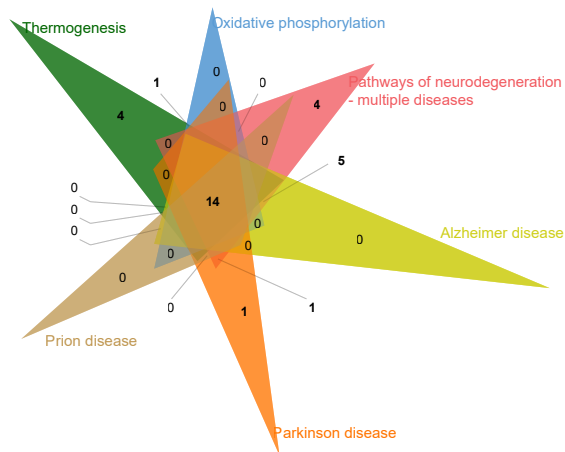

D

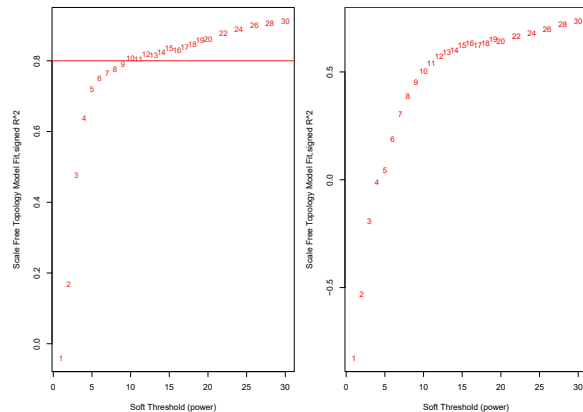

Supplement: giaf019_Supplemental_File [file giaf019_supplemental_file.zip › FigureS2_revision2.pdf]

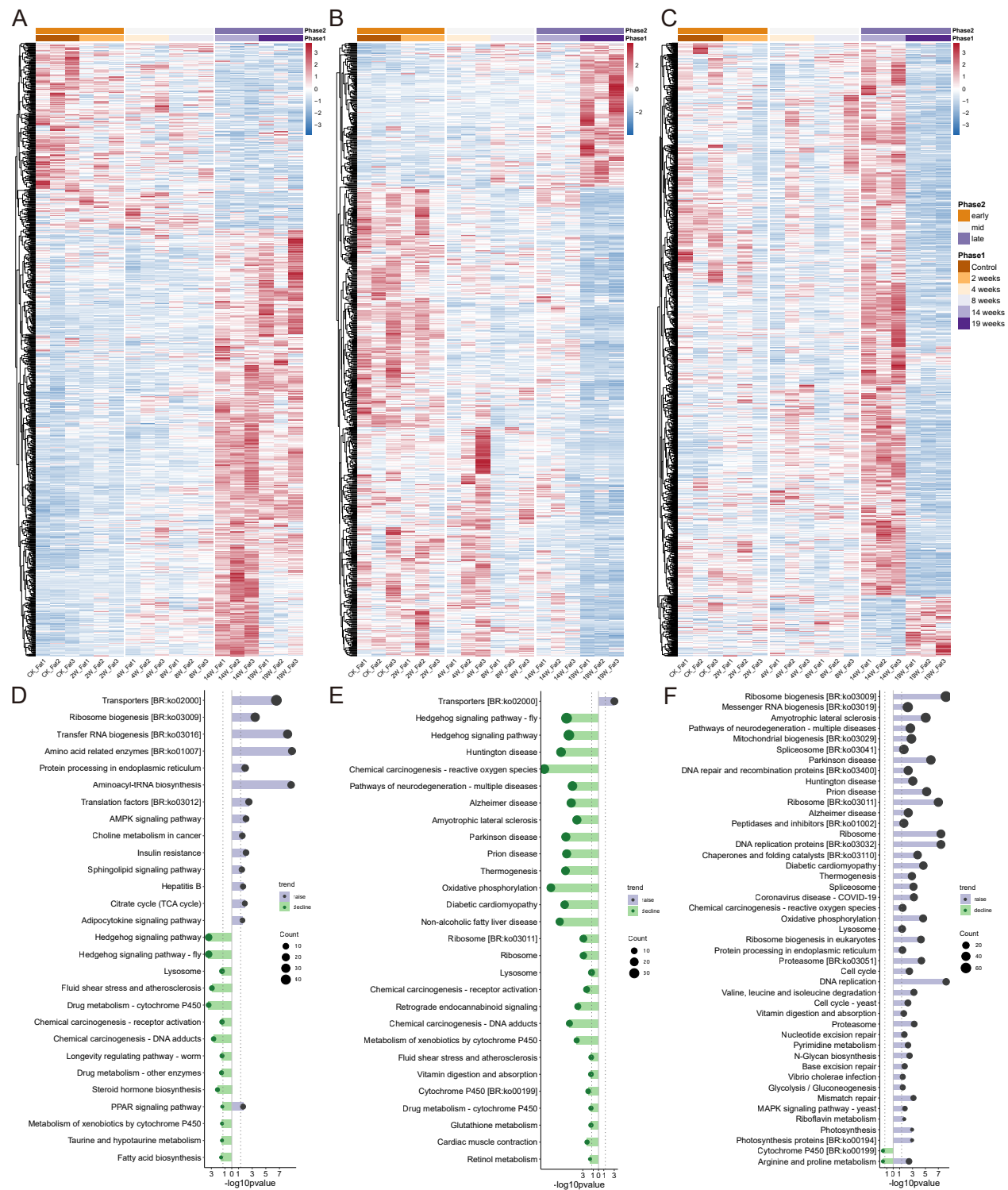

Supplement: giaf019_Supplemental_File [file giaf019_supplemental_file.zip › FigureS4_revision2.pdf]

A

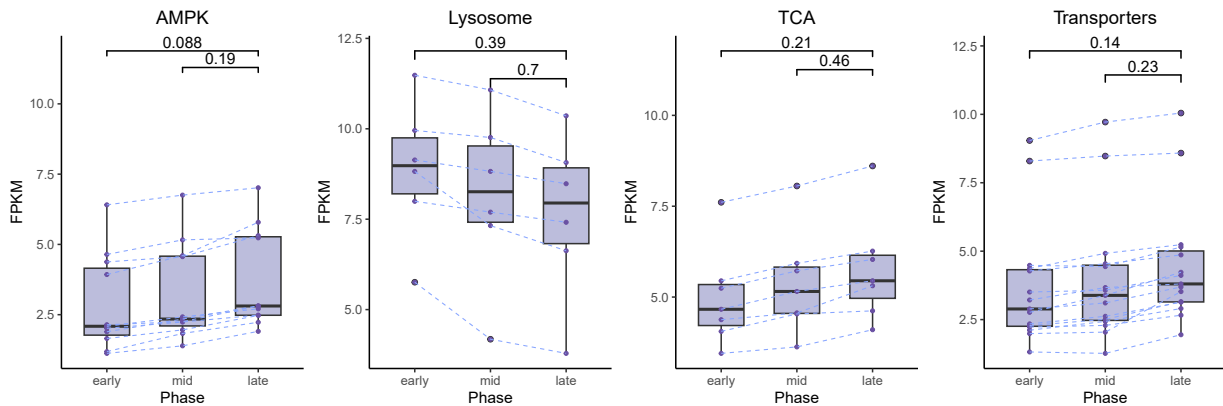

B

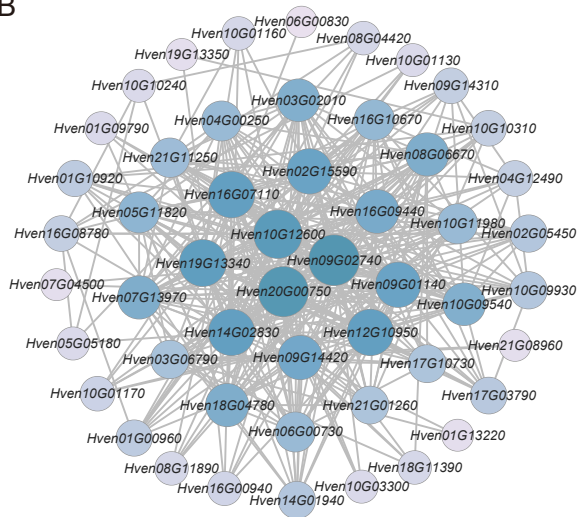

C

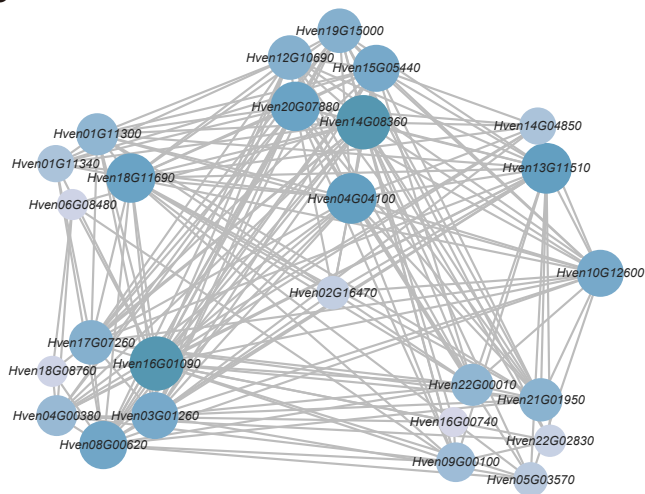

Supplement: giaf019_Supplemental_File [file giaf019_supplemental_file.zip › FigureS5_revision2.pdf]

A

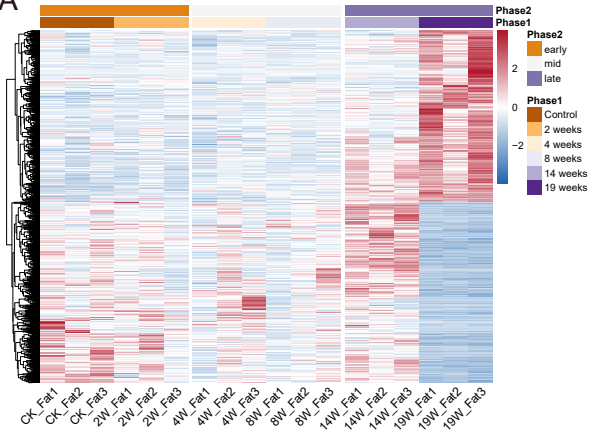

B

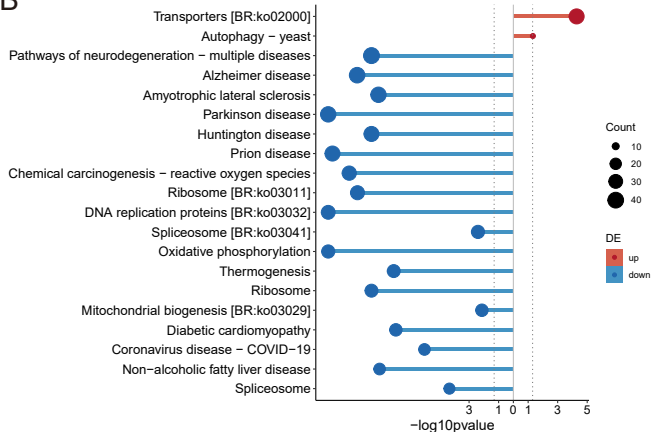

Supplement: giaf019_Supplemental_File [file giaf019_supplemental_file.zip › FigureS7_revision2.pdf]

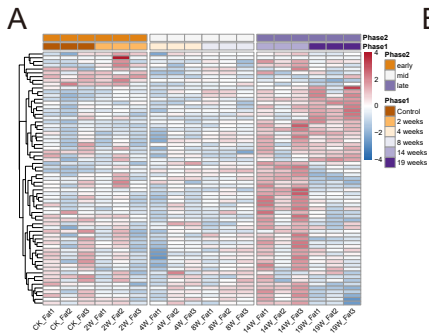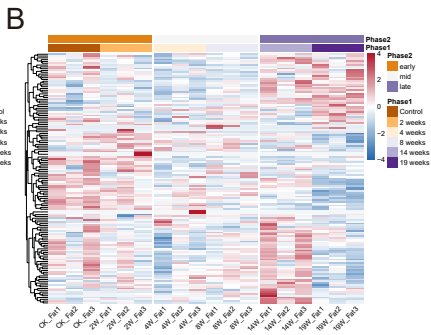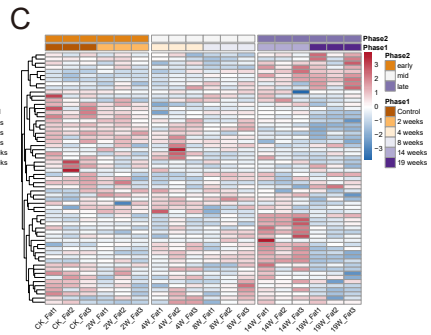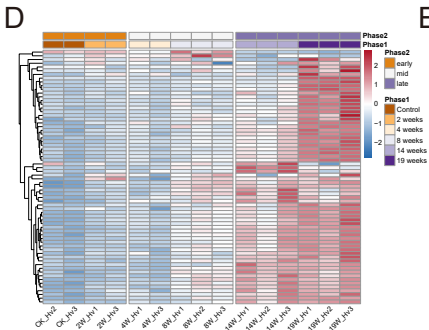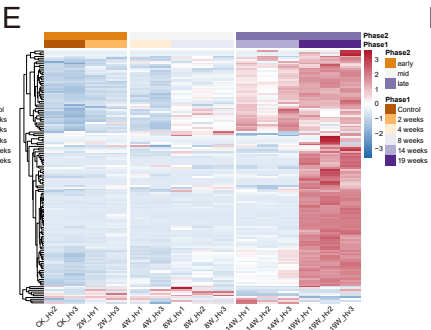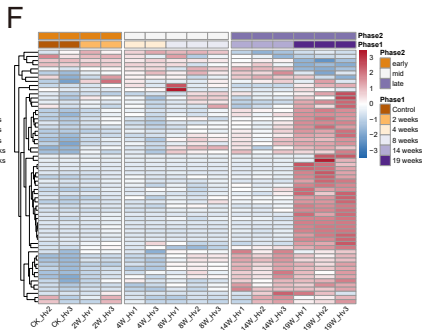

Supplement: giaf019_Supplemental_File [file giaf019_supplemental_file.zip › FigureS8_revision2.pdf]

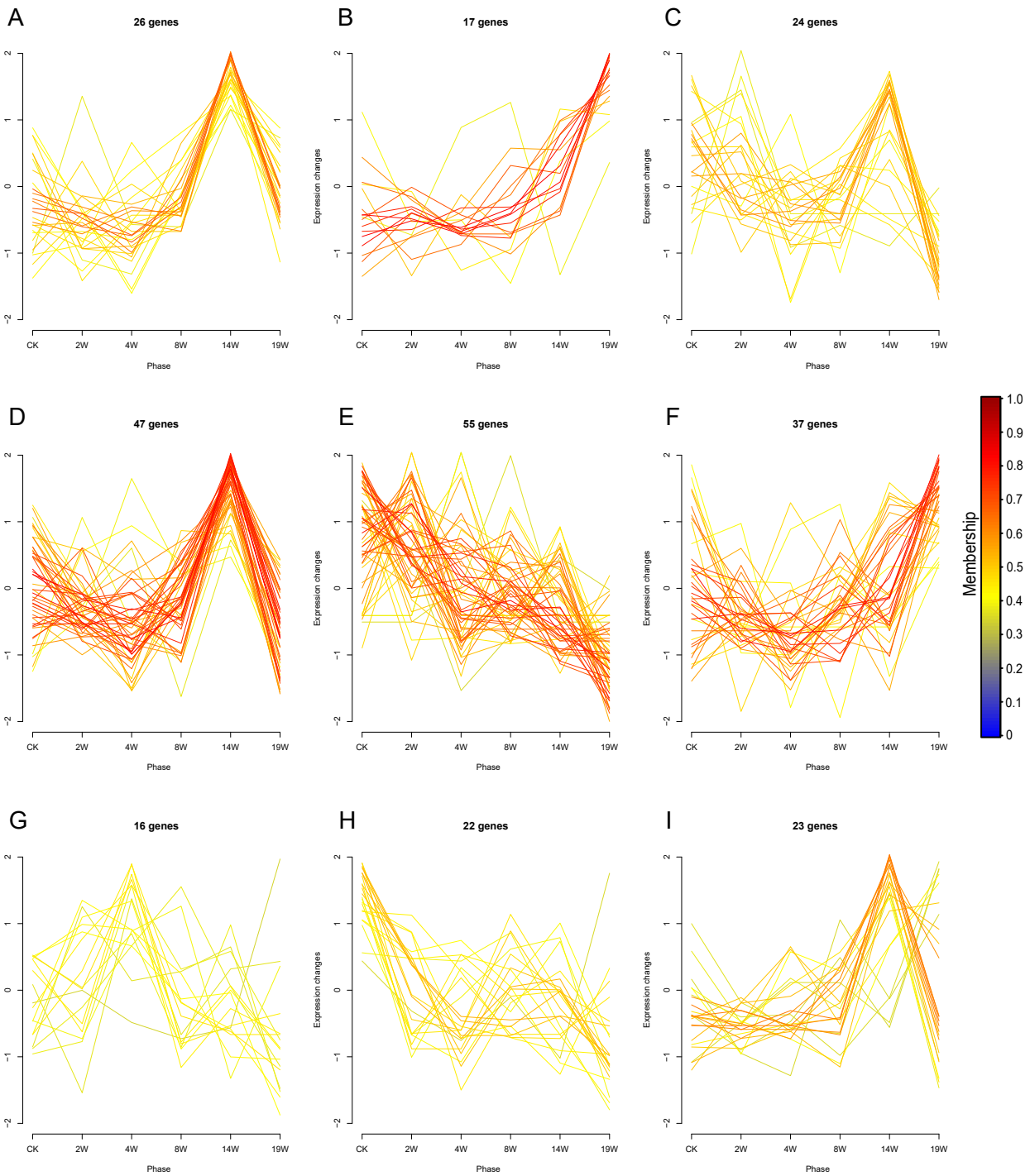

Supplement: giaf019_Supplemental_File [file giaf019_supplemental_file.zip › FigureS9_revision2.pdf]
