# Supplementary material for: Genomic and transcriptomic analyses of Heteropoda venatoria reveal the expansion of P450 family for starvation resistance in spiders
Source: Gigascience. 2025 Mar 21;14:giaf019. doi: 10.1093/gigascience/giaf019 (PMC11927401; doi:10.1093/gigascience/giaf019)
Supplement: giaf019_Response_to_Reviewer_Comments_Original_Submission [file giaf019_response_to_reviewer_comments_original_submission.pdf]

Author's Response To Reviewer Comments

Dear Editor,
First and foremost, we sincerely thank you for giving us the opportunity to revise our manuscript (GIGA-D-24-00314). The reviewers' comments have been invaluable and have greatly enhanced the quality of our article. We have carefully and thoroughly reviewed the feedback provided by the two esteemed reviewers and have made comprehensive revisions to the manuscript in accordance with their suggestions. We have also updated the data for some figures (Fig 1F, Fig 4B and Fig 4C) on the temporary private server of GigaDB. We hope that the updated manuscript will be accepted by GigaScience. Thank you once again for your excellent editorial work. The point-by-point responses to the reviewers' comments are presented below.

Reviewer reports:
Reviewer #1: In this study, the authors deciphered the chromosome-level genome of a RTA spider Heteropoda venatoria with large body size and generated comprehensive comparative transcriptomes of fat body and whole body among CK and starvation status. Generally, this study added important genomic and transcriptomic data of spiders and provided some cues in understanding the molecular changes during starvation. However, the organization of the manuscript is quite problematic.
R: Thank you very much for your overall positive evaluation of our manuscript. In this revision, we removed inferences and hypotheses from the results section as much as possible and readdressed them in the discussion. We also corrected some unreasonable aspects of the results. For example, in the differential transcriptomic analysis during the early, middle, and late stages of starvation, we deleted redundant inferences such as "These findings indicate that during the early starvation stage, energy metabolism in H. venatoria occurs regularly, with a sufficient supply of energy." and "This is also indicated by the activation of the AMPK signaling pathway, insulin resistance, and the adipocytokine signaling pathway." Additionally, we provided further explanation for potentially confusing areas in the results. For example, the statement "Interestingly, pathways related to neurodegeneration were also upregulated. An overlap analysis of genes in these pathways revealed that most genes related to neurodegenerative pathways are also involved in oxidative phosphorylation and thermogenesis (Supplementary Fig. S2C), which provides an explanation for why seemingly unreasonable pathways might exist during the early starvation stage in H. venatoria. All the revised sections in the text have been highlighted in red.

1. As to the Results section, please be concise and highlight the main results, avoiding accumulating complex results. Do not present too many statements in terms of introduction and discussion in Results. Do not raise too many hypotheses in the results.
R: Thank you for the comment. We have streamlined the results section and moved portions containing inferences and hypotheses to the discussion section. For example, in the section on the high-quality genome assembly and annotation of H. venatoria, we removed statements such as "These findings suggest that H. venatoria possesses a large genome with high repeat content and heterozygosity, presenting challenges in genome assembly"; "We speculate that during the evolution of spider genomes, two types of repeat sequences, TcMar and LTR sequences, had a significant impact on the size of spider genomes"; and "We speculate that these repeat sequences, which are rich in GC, are subjected to selective pressure owing to their structural stability or stronger binding to specific proteins, leading to an increase in the GC content in the repeat sequence regions". Similarly, other sections with redundant speculations or hypothetical content were also removed.

2. As for the involvement of the Hippo signaling pathway in lipid metabolism regulation, the cited literature and mentioned genes are not related to the results of this study. As for the analysis of P450 results, the descriptions of structural analysis are quite complex and difficult to understand. The authors did not explain clearly the relationship between the expansion of P450 genes and hunger resistance in the results of this study.
R: Thank you for the comment. Owing to an oversight in our work, while we mentioned the YAP gene in the text, we did not specify its identity in H. venatoria. In fact, in our KEGG annotation results for H. venatoria, we identified the YAP gene (Hven08G09310). We have revised the Results section to describe this more accurately: "Interestingly, while the Hippo pathway was upregulated at this stage, the key gene YAP

(Hven08G09310) was downregulated only in the Hippo pathway during the middle starvation period (Supplementary Table S7)." Additionally, we have moved some inferences about the Hippo pathway to the first paragraph of the discussion section.
Regarding the P450 section, we have simplified the description of the structural parts. For detailed information, please refer to the “Conserved domains in P450s” section. With respect to the relationship between P450 and starvation tolerance, the P450 family in H. venatoria has undergone significant expansion, particularly with clustered copies of the CYP3 genes. Additionally, P450 genes in H. venatoria are expressed primarily in the fat body. Current research indicates that P450 genes can lower metabolic rates or participate in lipid regulation. Therefore, we hypothesize that the expansion of the P450 family in H. venatoria enables it to maintain bodily functions at a lower metabolic rate during food scarcity, thereby extending its starvation tolerance. To clarify the relationship between P450 and starvation tolerance, we added the following description to the discussion section: “H. venatoria shows the most significant expansion, with 25 clustered copies of the CYP3 family on Chr4, highlighting the crucial role this expansion plays in its survival in complex environments. This expansion significantly enhances the starvation tolerance of H. venatoria, making it more adept at coping with environmental changes and stresses. Moreover, the large number of gene copies increases redundancy, thereby protecting H. venatoria from the effects of harmful mutations. If one gene copy loses its function, other copies can still perform the necessary functions.”

3. The author's analyses of DEG enrichment results in transcriptome analysis is confusing. Firstly,I can't agree with the authors in that "During the early stage of starvation (from CK to 2 W), many genes, specifically those involved in oxidative phosphorylation and thermogenesis pathways, were up-regulated (Fig. 2E). These findings indicate that during the early starvation stage, energy metabolism in H. venatoria occurs regularly, with sufficient supply of energy." There are a batch of DEGs between 2W and CK, and a lot of pathways involved in neurodegeneration related pathways. How to explain these changes? Secondly, as to 4W to 8W, I can not understand the relationship of down-regulation of hippo signaling pathway to the authors' speculation that "H. venatoria may reduce its cellular glucose uptake and utilization to adjust to the food-scarce environment.", as this pathway involved in lipid metabolism, as the authors stated. Thirdly, from 14 W to 19 W, pathways such Lysosome and apoptosis were down-regulated instead of up-regulated. So how the authors thought autophagy became more active?
R: Thank you for the comment. First, while there are indeed many pathways related to neurodegeneration, a key issue is the significant overlap between these pathways and those enriched with energy-related genes. These findings suggest that the genes whose expression was upregulated in the early starvation phase are associated mainly with energy metabolism, specifically thermogenesis and oxidative phosphorylation. To illustrate this more clearly, we have included a relevant Venn diagram (Supplementary Fig. S2C). Second, after reviewing the current research on the Hippo signaling pathway, we found no strong evidence to support a specific connection to glucose metabolism. Therefore, we have removed the hypothesis related to glucose metabolism. Third, on the basis of the fat analysis results, the lysosome and apoptosis pathways were indeed downregulated in the late starvation stage (Fig 2G). However, in the overall transcriptome of H. venatoria, particularly at the 19 W stage, the lysosome pathway was significantly upregulated. Consequently, we have revised the relevant descriptions and hypotheses accordingly.

4. "We speculate that during the evolution of spider genomes, two types of repeat sequences, TcMar and LTR sequences, had a significant impact on the size of spider genomes. Interestingly, we found that in H. venatoria chromosomes, regions with a high proportion of repeats also presented an increase in GC content (Fig. 1B)" The author's conclusion that high repeat region has higher CG content is based on Fig1B alone, which is too arbitrary. They need more solid evidence and more detailed analysis. For example, the GC content of TE region could be compared with that of whole genome, and the GC content of gene region. The significance of the relevant results should be explained. In addition, the author should make a more convincing discussion of this result based on the more literature.
R: Thank you for the comment. To provide a clearer comparison of the GC content between repeat and non-repeat regions, we conducted statistical analyses and visualizations of the GC content for non-repeat regions, repeat regions, LTR regions, and TcMar regions (Supplementary Fig. S1C and Supplementary Table S6). We also supplemented the results with a description of the repeat sequences. As shown in Fig. S1C, the GC content of the repeat regions was noticeably greater than that of non-repeat regions, which was statistically significant. Among the main types of repeat elements in H. venatoria, both the LTR and

TcMar regions also presented significantly higher GC contents than non-repeat regions, with TcMar having the highest GC proportion. Considering that this study focuses primarily on the starvation resistance of H. venatoria, and after extensive searches, we found almost no literature on the relationship between GC content and repeats. Therefore, we believe this area requires further exploration and research, and we are inclined to study spider repeats as a separate topic.

5. "We gathered genomic data and annotations for one scorpion and seven chromosome-level spider genomes using the scorpion as an outgroup [35-42]". Many spider genomes have been published at the chromosomal level. What were the principles behind the spider genomes the authors selected in this study?
R: Thank you for the comment. Although many chromosome-level spider genomes have been published, there are two chromosome-level genomes available for each of the four families: Uloboridae, Nephilidae, Linyphiidae and Theridiidae. We selected one genome from each family to represent it. This approach results in the loss of some species data; consequently, we added the remaining chromosome-level genomes from these four families to reconstruct the phylogenetic tree. The latest results can be seen in Fig. 1F.

6. "Transcriptome design for starvation resistance in H. venatoria" in Results should be partially moved Methods and here the authors should straightforwardly highlighted the results.
R: Thank you for the comment. Detailed descriptions of the methods have been removed from the Results section and are now thoroughly detailed in the Methods section.

7. I can't understand the significance of Fig 2C. The authors did not explain it in the manuscript, either.
R: Thank you for the comment. In our latest results, we have added a description regarding Fig. 2C: Fig. 2C and Supplementary Fig. S2B illustrate the expression correlation of transcriptomes from the fat body and whole body at different stages. These figures indicate that the transcriptomes presented the highest correlation at the 19-week stage. Consequently, we conducted a differential analysis of the transcriptome at 19 weeks.

8. "The PCA results from both the fat body and whole-body transcriptomes indicated that H. venatoria transcriptome at 19 weeks of starvation was markedly distinct from that at other stages (Fig. 2A, B). Consequently, we conducted a differential analysis of the transcriptome at 19 weeks." Please clarify how the comparative transcriptomes were conducted for differential analysis.
R: Thank you for the comment. We have provided a detailed description of the differential transcriptome analysis method used to compare the 19-week stage with other stages in the second paragraph of the Transcriptome Analysis section in the Methods section. Additionally, we adopted this approach because the differences in gene expression among these five groups of samples, from CK to 14 W, are quite significant. If we were to calculate differential genes by treating these five groups as a single control group, the resulting "differential genes" would likely be less accurate. Therefore, it is more reasonable to calculate differential genes for each group separately and then take the intersection.

9. The language should be polished.
R: Thank you for the comment. We have revised and refined the manuscript. The manuscript was polished by American Journal Experts.

Reviewer #2: The manuscript "Genomic and transcriptomic analyses of Heteropoda venatoria reveal the expansion of P450 family for starvation resistance in spider" uses comparative genomics to study the underlying mechanisms of starvation resistance. I appreciate that the authors have produced a high-quality genome for an RTA species. The methods are sound and some interesting gene families are highlighted as key factors in starvation resistance.
R: Thank you very much.

One primary concern I have relates to the study's setup and hypothesis. As currently written, the study comes across as a fishing expedition rather than a focused research project. Although the introduction is informative, it lacks a clear rationale for including this particular species. The reasoning only becomes apparent at the end of the gene family expansion and contraction section. Additionally, I am unsure if

being an active hunter makes feeding more unpredictable compared to web-based prey capture. I recommend incorporating this information into the introductory paragraph to better establish the context for the analysis.
R: Thank you for the comment. H. venatoria is a commonly found hunting spider that occurs indoors. Through long-term observation, we discovered that H. venatoria exhibits remarkable starvation endurance, which prompted us to design a transcriptome experiment to investigate the mechanisms underlying this ability. Since current research on spiders has focused primarily on silk and venom, there are almost no studies or reports on starvation endurance. This made it difficult to provide an extensive background on spider starvation endurance in the introduction. However, to clearly present the rationale for studying the starvation endurance of H. venatoria, we added the following statement to the introduction: "Our observations revealed that H. venatoria has an extraordinary ability to endure starvation and is capable of surviving for more than four months without food as long as the humidity is maintained. However, there is very little research on the starvation endurance of spiders." This statement indicates that our own observations led us to investigate the strong starvation endurance of H. venatoria. Additionally, as there is no current research comparing the capture efficiency between web-building and hunting spiders, we have decided to remove that description for rigorous language.

While terms like "autophagy" and "energy homeostasis" are appropriate for a scientific audience, consider briefly defining them for clarity, especially if the intended audience might not be familiar with all the terminology. Although authors mention that there is no high-quality genome sequence for H. venatoria, it could be helpful to elaborate on why this is significant for understanding starvation resistance. A brief explanation of how genomic data could enhance understanding of the molecular mechanisms involved would strengthen this point. The conclusion provides a clear goal for your study, but it could be more impactful. You might want to emphasize the broader implications of your research findings for ecological conservation and biodiversity. End with a statement about the importance of understanding these mechanisms in the context of preserving ecosystems and addressing challenges posed by climate change.
R: Thank you for the comment. In the introduction of the article, we have added explanations regarding "autophagy" and "energy homeostasis". Energy homeostasis is achieved by balancing energy expenditure and energy intake. Cellular autophagy is a self-degradative process that is equally crucial for maintaining energy homeostasis during periods of starvation. We have also included a description of how the genome can help us understand the mechanisms related to starvation resistance. A high-quality genome sequence is crucial for understanding how H. venatoria regulates its metabolism and survival under starvation conditions. With a genome, we can more effectively identify key genes and regulatory elements, thereby revealing genes and molecular pathways associated with starvation resistance. This helps us gain a deeper understanding of the underlying mechanisms involved. Finally, we emphasize that multi-omics studies on starvation resistance can provide a scientific basis for the conservation of biodiversity and the maintenance of ecosystem functions.

For the discussion, while the content is detailed, some parts feel slightly repetitive or could be more concise. For instance, the description of P450 gene expression could be streamlined by removing redundant mentions of their role in metabolic rate regulation. Example: In the discussion section "Interestingly, we found that some P450 families are expanded in H. venatoria, and most P450 genes are more highly expressed in the fat body than in other tissues…" This point is later reiterated in the sentence about other spider species. These ideas could be combined for efficiency.
R: Thank you for the comment. We have refined the discussion section by consolidating redundant paragraphs and removing unnecessary phrases. For example, we integrated the higher expression of P450 families in the fat body across various spiders "P450 families play an important role in the fat body, with most P450 genes showing higher expression in this tissue than in other tissues. This pattern was observed not only in H. venatoria and T. clavata (Fig. 5B and supplementary Fig. S10) but also in P. pseudoannulata [51]." We also removed the redundant paragraph "In addition to being highly expressed in the fat body in H. venatoria, P450 genes are also predominantly expressed in fat bodies in P. pseudoannulata and T. clavata. These findings indicate that the role of P450 genes in the inhibition of metabolic rates is a common phenomenon in spiders, not only in H. venatoria. Owing to the well-developed P450 families in spiders and to their dormancy habit [10], spiders have a low metabolic rate, allowing them to endure long periods of starvation, with the expansion of P450 families in H. venatoria resulting in starvation resistance."

The paragraph about the phylogenetic analysis of the CYP3 clan could be shortened. While it is an

interesting finding, some of the details (like the number of genes or proteins) might be better suited for the main text rather than a summary. Focusing more on the functional implications of these duplications would keep the reader engaged. Though the findings are well-explained, the broader significance could be emphasized more explicitly. For example, why is understanding these mechanisms important for the field of arachnid biology, evolutionary biology, or even practical applications (e.g., pest control, conservation)? You could add a closing sentence that ties everything together and highlights the broader relevance of the findings, such as the evolutionary or ecological importance of these adaptations in spiders.
R: Thank you for the comment. We shortened the section on the phylogeny of the CYP3 family, with the final revision: “The phylogenetic tree for the CYP3 clan across these spiders revealed that 25 CYP3 clan genes from H. venatoria (representing 27 proteins) are located within a region of less than 2.5 Mb on Chr4 and cluster on the same branch (Fig. 4B).” Additionally, we added details about the number of P450 genes: “Among these, H. venatoria has the greatest number of P450 genes, totaling 141, whereas P. pseudoannulata, which is also part of the RTA clade, has only 82 P450 genes.” Finally, we added “These gene expansions likely reflect enhanced environmental adaptability in spiders, potentially influencing their metabolic capabilities, predation strategies, or ecological adaptations” to highlight the significance of CYP3 expansion.

Other comments:
Last paragraph of the introduction: When introducing Heteropoda venatoria, please spell out the species name the first time that is used. The sentence "However, these findings indicate that H. venatoria does not feed in a stable manner and often experiences periods of starvation." Does not fit the rest of the text. Finding from what study?
R: Thank you for the comment. We have revised the introduction to use the full name “Heteropoda venatoria” for the first time. Regarding the sentence, "However, these findings indicate that H. venatoria does not feed in a stable manner and often experiences periods of starvation," we agree that it was imprecise. We have removed this sentence and restructured the paragraph for clarity.

Transcription design for starvation resistance in H. venatoria section:
First sentence: What samples? confusing to start like this. Please add information about the samples. You could delete "the samples of H. venatoria were subjected to" it will read better.
R: Thank you for the comment. We have deleted "the samples of H. venatoria were subjected to."

Are all 23 CYP# clan genes on chromosome 4 tandemly arrayed?
R: After reanalysis, we found that 25 CYP clan genes are arranged in tandem.

Figure 4 - add more information about the figure. For pannel C, What do the red lines show? Grey? Numbers in the circles? While I know what they represent, other readers might not.
R: Thank you for the comment. We have added this information to Figure 4C.

The finding that H. venatoria chromosomes have undergone lots of chromosomal fragmentation is very interesting, and it is clearly shown on the figure. Which is why I think that more detail is needed.
R: Thank you for the comment. In fact, the phenomenon of chromosomal breakage and fusion is quite common in spiders, and it is indeed a very interesting point. We prefer to study this point as a separate topic, and since this topic is not the main focus of this section, we have not analyzed it in great detail.

In this sentence "In Uloborus diversus, members of this subfamily are located on Chr5 and an unanchored scaffold." You need to specify which members.
R: Thank you for the comment. We have labeled the names of the relevant members.

Figure 5 - Include a description of the tissues. What is Epi? Ducts? Tail?
R: Thank you for the comment. We have added the meanings of the sample abbreviations to the annotations in Figure 5.
