# Supplementary material for: Genomic and transcriptomic analyses of Heteropoda venatoria reveal the expansion of P450 family for starvation resistance in spiders
Source: Gigascience. 2025 Mar 21;14:giaf019. doi: 10.1093/gigascience/giaf019 (PMC11927401; doi:10.1093/gigascience/giaf019)
Supplement: giaf019_Response_to_Reviewer_Comments_Revision_1 [file giaf019_response_to_reviewer_comments_revision_1.pdf]

Author's Response To Reviewer Comments

RESPONSE TO REVIEWER COMMENTS:
We thank the Reviewers for the time and effort spent in carefully reviewing our manuscript and providing constructive comments. Point-by-point responses to all comments and modifications to the manuscript are listed below.

Reviewer #1: Although I'm not convinced by the authors explanation on expression changes of lysosome pathway and the apoptosis pathway, or the functional significance of expansion of P450 genes, I think the datasets and the authors discussion of the genomic and transcriptomic features of the wandering spider in response to starvation are very important for further studies on this highly derived spider group.
R: We thank the reviewer for the time and effort that you have put into reviewing the previous version of our manuscript. We appreciate the detailed and constructive comments. Those comments are all valuable and very helpful for revising and improving our paper, as well as important for guiding our research. We have studied the comments carefully and made corrections, which we hope will meet with approval.

1. The authors may need to clarify the relationship of lysosome pathway, the autophagy pathway, and the apoptosis pathway. Factually, they did not observe expressional changes of genes in the autophagy pathway. So it is difficult to highlight role of autophagy in response to starvation and energy supply.
R: We thank the Reviewer for the comments and agree with the suggestions. To clarify the relationships among the gene expression trends of the autophagy, lysosome and apoptosis pathways at different stages of starvation resistance, we extracted the expression matrices of all genes annotated to these three pathways from the fat body and whole-body transcriptomes. In the fat body, the expression of most genes in the three pathways was relatively low in the early and middle stages, peaked at 14 W, and then sharply declined at 19 W (Supplementary Fig. S9A, C, D, and I). In the whole-body, most genes presented low expression levels during the early and middle stages of starvation, followed by a sharp increase beginning at 14W, or a gradual increase starting from the middle stage and persisting through the late stage of starvation (Supplementary Fig. S10A, C, D, E, H, and I). We speculate that, as the primary energy-supplying organ in H. venatoria, the fat body was nearly fully depleted of its energy reserves through normal oxidative metabolism by 14 W. At this stage, the fat body could only rely on autophagy and apoptosis to process its remaining tissue. This explains why many genes in these pathways reached their peak expression levels at this time. However, energy production through these processes in the fat body alone was insufficient to sustain an organism’s overall energy demands. Consequently, autophagy and related responses in other tissues of H. venatoria became increasingly active and continued to intensify until 19 W. At this time, the fat body was essentially incapable of supplying energy, forcing most tissues in H. venatoria to depend on autophagy and apoptosis pathways for energy production, leading to the peak activity of these three pathways. Moreover, a recently published paper revealed that prolonged starvation promotes both autophagy and apoptosis in silkworms (1).
Overall, we believe that starvation resistance is closely related to these three pathways. On the basis of the above content, we have revised the Results and Discussion sections of the manuscript and highlighted them in red.

(1) Cheng J, Qin X, Han B, et al. Mechanism of starvation induced autophagy and apoptosis in the midgut of silkworm, Bombyx mori, based on calcium homeostasis. Insect Mol Biol. 2024; https://doi.org/10.1111/imb.12981.
