# Supplementary material for: Genomic and transcriptomic analyses of Heteropoda venatoria reveal the expansion of P450 family for starvation resistance in spiders
Source: Gigascience. 2025 Mar 21;14:giaf019. doi: 10.1093/gigascience/giaf019 (PMC11927401; doi:10.1093/gigascience/giaf019)
Supplement: giaf019_Reviewer_1_Report_Original_Submission — Hui Xiang, PH.D -- 9/17/2024 [file giaf019_reviewer_1_report_original_submission.pdf]

Reviewer Report

Title: Genomic and transcriptomic analyses of Heteropoda venatoria reveal the expansion of P450 family for starvation resistance in spider

Version: Original Submission **Date:** 9/17/2024

Reviewer name: Hui Xiang, PH.D

Reviewer Comments to Author:

In this study, the authors deciphered the chromosome-level genome of a RTA spider Heteropoda venatoria with large body size and generated comprehensive comparative transcriptomes of fat body and whole body among CK and starvation status. Generally, this study added important genomic and transcriptomic data of spiders and provided some cues in understanding the molecular changes during starvation. However, the organization of the manuscript is quite problematic.

1. As to the Results section, please be concise and highlight the main resultsï¼Œavoiding accumulating complex results. Do not present too many statements in terms of introduction and discussion in Results. Do not raise too many hypotheses in the results.
2. As for the involvement of the Hippo signaling pathway in lipid metabolism regulation, the cited literature and mentioned genes are not related to the results of this study. As for the analysis of P450 results, the descriptions of structural analysis are quite complex and difficult to understand. The authors did not explain clearly the relationship between the expansion of P450 genes and hunger resistance in the results of this study.
3. The author's analyses of DEG enrichment results in transcriptome analysis is confusing. Firstly,I can't agree with the authors in that "During the early stage of starvation (from CK to 2 W), many genes, specifically those involved in oxidative phosphorylation and thermogenesis pathways, were up-regulated (Fig. 2E). These findings indicate that during the early starvation stage, energy metabolism in H. venatoria occurs regularly, with sufficient supply of energy." There are a batch of DEGs between 2W and CK, and a lot of pathways involved in neurodegeneration related pathways. How to explain these changes? Secondly, as to 4W to 8W, I can not understand the relationship of down-regulation of hippo signaling pathway to the authors' speculation that "H. venatoria may reduce its cellular glucose uptake and utilization to adjust to the food-scarce environment.", as this pathway involved in lipid metabolism, as the authors stated. Thirdly, from 14 W to 19 W, pathways such Lysosome and apoptosis were down-regulated instead of up-regulated. So how the authors thought autophagy became more active?
4. "We speculate that during the evolution of spider genomes, two types of repeat sequences, TcMar and LTR sequences, had a significant impact on the size of spider genomes. Interestingly, we found that in H. venatoria chromosomes, regions with a high proportion of repeats also presented an increase in GC content (Fig. 1B)" The author's conclusion that high repeat region has higher CG content is based on Fig1B alone, which is too arbitrary. They needs more solid evidence and more detailed analysis. For example, the GC content of TE region could be compared with that of whole genome, and the GC content of gene region. The significance of the relevant results should be explained. In addition, the author should make a more convincing discussion of this result based on the more literature.

5. "We gathered genomic data and annotations for one scorpion and seven chromosome-level spider genomes using the scorpion as an outgroup [35-42]"ã€,Many spider genomes have been published at the chromosomal level. What were the principles behind the spider genomes the authors selected in this study?
6. "Transcriptome design for starvation resistance in H. venatoria" in Results should be partially moved Methods and here the authors should straightforwardly highlighted the results .
7. I can't understand the significance of Fig 2C. The authors did not explain it in the manuscript, either.
8. "The PCA results from both the fat body and whole-body transcriptomes indicated that H. venatoria transcriptome at 19 weeks of starvation was markedly distinct from that at other stages (Fig. 2A, B). Consequently, we conducted a differential analysis of the transcriptome at 19 weeks." Please clarify how the comparative transcriptomes were conducted for differential analysis.
9. The language should be polished.

Methods

Are the methods appropriate to the aims of the study, are they well described, and are necessary controls included? Choose an item.

Conclusions

Are the conclusions adequately supported by the data shown? Choose an item.

Reporting Standards

Does the manuscript adhere to the journal’s guidelines on minimum standards of reporting? Choose an item.

Choose an item.

Statistics

Are you able to assess all statistics in the manuscript, including the appropriateness of statistical tests used? Choose an item.

Quality of Written English

Please indicate the quality of language in the manuscript: Choose an item.

Declaration of Competing Interests

Please complete a declaration of competing interests, considering the following questions:

- Have you in the past five years received reimbursements, fees, funding, or salary from an organisation that may in any way gain or lose financially from the publication of this manuscript, either now or in the future?
- Do you hold any stocks or shares in an organisation that may in any way gain or lose financially from the publication of this manuscript, either now or in the future?
- Do you hold or are you currently applying for any patents relating to the content of the manuscript?
- Have you received reimbursements, fees, funding, or salary from an organization that holds or has applied for patents relating to the content of the manuscript?
- Do you have any other financial competing interests?
- Do you have any non-financial competing interests in relation to this paper?

If you can answer no to all of the above, write 'I declare that I have no competing interests' below. If your reply is yes to any, please give details below.

I declare that I have no competing interests.

I agree to the open peer review policy of the journal. I understand that my name will be included on my report to the authors and, if the manuscript is accepted for publication, my named report including any attachments I upload will be posted on the website along with the authors' responses. I agree for my report to be made available under an Open Access Creative Commons CC-BY license (http://creativecommons.org/licenses/by/4.0/). I understand that any comments which I do not wish to be included in my named report can be included as confidential comments to the editors, which will not be published.

Choose an item.

To further support our reviewers, we have joined with Publons, where you can gain additional credit to further highlight your hard work (see: https://publons.com/journal/530/gigascience). On publication of this paper, your review will be automatically added to Publons, you can then choose whether or not to claim your Publons credit. I understand this statement.

Yes Choose an item.
