# Supplementary material for: Genomic and transcriptomic analyses of Heteropoda venatoria reveal the expansion of P450 family for starvation resistance in spiders
Source: Gigascience. 2025 Mar 21;14:giaf019. doi: 10.1093/gigascience/giaf019 (PMC11927401; doi:10.1093/gigascience/giaf019)
Supplement: giaf019_Reviewer_2_Report_Original_Submission — Sandra Correa-Garhwal -- 9/27/2024 [file giaf019_reviewer_2_report_original_submission.pdf]

Reviewer Report

Title: Genomic and transcriptomic analyses of Heteropoda venatoria reveal the expansion of P450 family for starvation resistance in spider

Version: Original Submission **Date:** 9/27/2024

Reviewer name: Sandra Correa-Garhwal

Reviewer Comments to Author:

The manuscript "Genomic and transcriptomic analyses of Heteropoda venatoria reveal the expansion of P450 family for starvation resistance in spider" uses comparative genomics to study the underlying mechanisms of starvation resistance. I appreciate that the authors have produced a high-quality genome for an RTA species. The methods are sound and some interesting gene families are highlighted as key factors in starvation resistance.

One primary concern I have relates to the study's setup and hypothesis. As currently written, the study comes across as a fishing expedition rather than a focused research project. Although the introduction is informative, it lacks a clear rationale for including this particular species. The reasoning only becomes apparent at the end of the gene family expansion and contraction section. Additionally, I am unsure if being an active hunter makes feeding more unpredictable compared to web-based prey capture. I recommend incorporating this information into the introductory paragraph to better establish the context for the analysis.

While terms like "autophagy" and "energy homeostasis" are appropriate for a scientific audience, consider briefly defining them for clarity, especially if the intended audience might not be familiar with all the terminology. Although authors mention that there is no high-quality genome sequence for H. venatoria, it could be helpful to elaborate on why this is significant for understanding starvation resistance. A brief explanation of how genomic data could enhance understanding of the molecular mechanisms involved would strengthen this point. The conclusion provides a clear goal for your study, but it could be more impactful. You might want to emphasize the broader implications of your research findings for ecological conservation and biodiversity. End with a statement about the importance of understanding these mechanisms in the context of preserving ecosystems and addressing challenges posed by climate change.

For the discussion, while the content is detailed, some parts feel slightly repetitive or could be more concise. For instance, the description of P450 gene expression could be streamlined by removing redundant mentions of their role in metabolic rate regulation. Example: In the discussion section "Interestingly, we found that some P450 families are expanded in H. venatoria, and most P450 genes are more highly expressed in the fat body than in other tissues…" This point is later reiterated in the sentence about other spider species. These ideas could be combined for efficiency.

The paragraph about the phylogenetic analysis of the CYP3 clan could be shortened. While it is an interesting finding, some of the details (like the number of genes or proteins) might be better suited for the main text rather than a summary. Focusing more on the functional implications of these duplications would keep the reader engaged. Though the findings are well-explained, the broader significance could

be emphasized more explicitly. For example, why is understanding these mechanisms important for the field of arachnid biology, evolutionary biology, or even practical applications (e.g., pest control, conservation)?
You could add a closing sentence that ties everything together and highlights the broader relevance of the findings, such as the evolutionary or ecological importance of these adaptations in spiders.
Other comments:
Last paragraph of the introduction: When introducing Heteropoda venatoria, please spell out the species name the first time that is used. The sentence "However, these findings indicate that H. venatoria does not feed in a stable manner and often experiences periods of starvation." Does not fit the rest of the text. Finding from what study?
Transcription design for starvation resistance in H. venatoria section:
First sentence: What samples? confusing to start like this. Please add information about the samples. You could delete "the samples of H. venatoria were subjected to" it will read better.
Are all 23 CYP# clan genes on chromosome 4 tandemly arrayed?
Figure 4 - add more information about the figure. For pannel C, What do the red lines show? Grey? Numbers in the circles? While I know what they represent, other readers might not. The finding that H. venatoria chromosomes have undergone lots of chromosomal fragmentation is very interesting, and it is clearly shown on the figure. Which is why I think that more detail is needed.
In this sentence "In Uloborus diversus, members of this subfamily are located on Chr5 and an unanchored scaffold." You need to specify which members.
Figure 5 - Include a description of the tissues. What is Epi? Ducts? Tail?

Methods

Are the methods appropriate to the aims of the study, are they well described, and are necessary controls included? Choose an item.

Conclusions

Are the conclusions adequately supported by the data shown? Choose an item.

Reporting Standards

Does the manuscript adhere to the journal's guidelines on minimum standards of reporting? Choose an item.

Choose an item.

Statistics

Are you able to assess all statistics in the manuscript, including the appropriateness of statistical tests used? Choose an item.

Quality of Written English

Please indicate the quality of language in the manuscript: Choose an item.

Declaration of Competing Interests

Please complete a declaration of competing interests, considering the following questions:

- Have you in the past five years received reimbursements, fees, funding, or salary from an organisation that may in any way gain or lose financially from the publication of this manuscript, either now or in the future?
- Do you hold any stocks or shares in an organisation that may in any way gain or lose financially from the publication of this manuscript, either now or in the future?
- Do you hold or are you currently applying for any patents relating to the content of the manuscript?
- Have you received reimbursements, fees, funding, or salary from an organization that holds or has applied for patents relating to the content of the manuscript?
- Do you have any other financial competing interests?
- Do you have any non-financial competing interests in relation to this paper?

If you can answer no to all of the above, write 'I declare that I have no competing interests' below. If your reply is yes to any, please give details below.

I have no competing interests

I agree to the open peer review policy of the journal. I understand that my name will be included on my report to the authors and, if the manuscript is accepted for publication, my named report including any attachments I upload will be posted on the website along with the authors' responses. I agree for my report to be made available under an Open Access Creative Commons CC-BY license (http://creativecommons.org/licenses/by/4.0/). I understand that any comments which I do not wish to be included in my named report can be included as confidential comments to the editors, which will not be published.

Choose an item.

To further support our reviewers, we have joined with Publons, where you can gain additional credit to further highlight your hard work (see: https://publons.com/journal/530/gigascience). On publication of this paper, your review will be automatically added to Publons, you can then choose whether or not to claim your Publons credit. I understand this statement.

Yes Choose an item.
